# Downscaled global 60-meter resolution estimates of irrigation water sources (2000–2015)

**DOI:** 10.1038/s41597-025-05920-x

**Published:** 2025-10-09

**Authors:** Fengwei Hung, Davide Danilo Chiarelli, James S. Famiglietti, Marc F. Müller

**Affiliations:** 1 Environmental Change Initiative, University of Notre Dame, Notre Dame, Indiana USA; 2 School of Sustainability, Arizona State University, Tempe, Arizona USA; 3 Department of Civil and Environmental Engineering, Politecnico di Milano, Milano, Italy; 4 Eawag, Swiss Federal Institute of Aquatic Science and Technology, Dübendorf, Switzerland

**Keywords:** Hydrology, Environmental sciences

## Abstract

This dataset provides high-resolution (60 m) global irrigation maps to support water resource and agricultural management. It identifies the likely irrigation status (rainfed or irrigated) and water source (groundwater or surface water) of croplands for 2000, 2005, 2010, and 2015. We downscaled a 10-km irrigation dataset derived from national and subnational statistics (GMIA) using (i) spatial patterns between high-resolution (30 m) cropland and nearby surface water, and (ii) irrigation water requirements from a global crop model. Validation used household agriculture surveys in India (N = 8,355) and a U.S. well database (N = 1,505,371). In the U.S., our method achieved 85% accuracy in distinguishing groundwater use within 2 km of wells – substantially higher than GMIA (25%). In India’s groundwater-dominated regions, our estimates performed comparably to GMIA (73% vs. 72%). These results suggest our dataset offers a more accurate and spatially detailed representation of irrigation water sources, enabling improved analysis of agricultural water use.

## Introduction and Background

Climate change and increasing human water demands have deepened water scarcity in arid and semiarid regions, accelerating the depletion of surface water and groundwater resources, such as Pakistan, northern China, Iran, Mexico and the Southwestern United States (U.S.)^1–3^. Agriculture significantly contributes to this issue, as 70% of global surface and groundwater extractions are used for irrigation^4^. Much of this water is used unsustainably^5^, meaning irrigation water requirements cannot be met without compromising environmental flows or depleting groundwater resources^6–8^. Unsustainable water use often leads to potential competition for water with the environment or other users, resulting in inefficient and inequitable water appropriation^9^. These effects are strongly influenced by regional climatic characteristics, the source of irrigation water and the specific location within the landscape from which the water is extracted. For example, a study in Khorasan Razavi (Iran) highlighted the impacts of groundwater extraction on local species and their ecological services^10^. Another study indicated that water extraction can affect aquatic habitat connectivity and cause various degrees of disturbance in ecosystem functions depending on the location of the extraction^10–12^. Similarly, the externalities and incentives for over-pumping groundwater are shaped by the spatial patterns of groundwater use and the geographic distance between adjacent well fields^13^. In many water-scarce regions of the world, these dynamics are unfolding within the context of an ongoing transition from traditional smallholder farming to large-scale commercial agriculture in low and middle income countries, resulting in dramatic changes to the landscape at a fine scale^14^. These changes underscore the need to monitor irrigated cropland at a high resolution and global scale.

Mapping the location of irrigated cropland and identifying the source of irrigation water (groundwater vs. surface water) has proven challenging to achieve consistently at high resolution and at a global scale^2,15^. Notable efforts have been made to generate high-resolution maps of irrigated cropland in specific countries, such as the U.S. (30 m resolution), India (250 m), and China (500 m), where detailed agricultural census data are available and supplemented by remote sensing imagery^16–18^. At the global scale, a series of studies has focused on collecting and harmonizing country-level agricultural census data to generate gridded estimates of global irrigated areas^15,19–21^. A first global inventory of water withdrawals from census data at national and subnational levels in the early 2000’s was combined with a global crop water use model to produce a first version of the Global Map of Irrigated Areas (GMIA dataset)^15,19^, with global estimates of surface areas equipped for irrigation (AEI) at 5-arcmin resolution (approximately 10 km at the equator). AEIs are defined as areas having access to infrastructure necessary for irrigation and differ from the areas actually irrigated, which depend on local climate and farming practices and can change from year to year, for example with crop rotation. The GMIA dataset has been updated multiple times over the past two decades^5,20,21^, with the latest update in 2024^5^ providing global AEI maps for the years of 2000, 2005, 2010, and 2015.

A key limitation of those datasets is their reliance on agricultural survey data, the quality of which can vary across countries and over time. Consequently, AEI estimates in some countries could have a substantially lower effective resolution than 5 arcmin, depending on the administrative units used in the national surveys. Several studies attempted to improve irrigation area mapping. For example, MIRCA2000^22^ is a collection of crop-specific irrigation maps, building on the GMIA dataset for the year 2000, and was incorporated into later versions of GMIA. Separately, multiple studies leverage the advancement of machine learning to improve temporal and spatial resolution of the global irrigation maps by combining agricultural census, remote sensing, and climate data^18,23,24^.

A notable effort to distinguish surface water from groundwater irrigation at the global scale is the study by Siebert *et al*.^2^, which incorporated information on irrigation water sources from national agricultural census datasets into the GMIA dataset, using a similar modelling approach and a data inventory compiled around 2005^2,21^. To the best of our knowledge, this remains the only global dataset focused specifically on groundwater irrigation^2,25,26^. However, the low spatial resolution of the GMIA limits its utility for effective water resources and policy research, particularly in contexts such as integrated and transboundary aquifer management where the location of pumping plays a critical role in triggering common-pool effects and driving premature depletion^13^.

To address the needs of fine-resolution global groundwater irrigation maps, we generate global maps of primary crop water sources (rainfall, groundwater and surface water irrigation) at 60-m resolution for 2000, 2005, 2010, and 2015. The algorithm to create the water source prediction involves two steps. First, we leverage two recent sources of information and a machine learning algorithm to downscale AEI estimates from the GMIA dataset:

- a high-resolution (30 m) estimate of global cropland extent^27^, which is used to identify the most likely source of irrigation given both the distance of each cropland pixel to the nearest source of surface water and the spatial texture of cropland patches.
- a global model of crop water use^9^ used to generate crop water deficit maps to constrain our downscaling procedure.

Next, each irrigated pixel of the downscaled images is assigned to one of the water sources categories using spatial distribution of surface water sources related to croplands and images of groundwater irrigation ratio derived from national and subnational statistics^2^. The water source prediction was validated against field-level data from rice plot survey in India^28^ and from a recent groundwater well database in the U.S.^29^. To assess data prediction accuracy, we calculated three accuracy metrics (user, producer and overall accuracies) at 300 m, 500 m, 1 km, 2 km, 3 km and 5 km.

## Methods

**Fig. 1:**
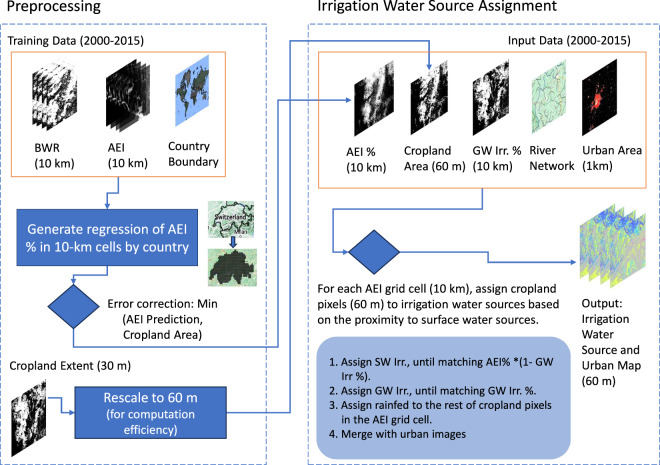
Data preprocessing and downscaling flowchart. The resolution of the images is indicated in the parenthesis.

We developed a downscaling algorithm to map rainfed, surface-water, and groundwater irrigation at 60-m resolution by integrating high-resolution cropland extent data with estimates of crop water requirements. As shown in Fig. 1, this algorithm proceeds in two major stages – data preprocessing and irrigation water source assignment. During preprocessing, we harmonize coarse-scale AEI estimates with high-resolution cropland distributions, adjusting for biophysical irrigation water requirements. We then assign irrigation water sources to each cropland pixel based on proximity to major surface water features, the fraction of groundwater irrigation from the GMIA, and the assumption that irrigation infrastructure tends to be spatially consolidated to minimize conveyance losses. Implemented on the Google Earth Engine (GEE) platform, this approach enables large-scale geospatial analysis and efficient handling of multi-temporal global datasets. Below, we describe each step in detail, along with the key data inputs, processes, and assumptions underlying the algorithm.

### Data Source

- **Irrigated area**. The GMIA AEI dataset^5,20^ contains gridded global AEI estimates for 2000, 2005, 2010, and 2015 at 5-arcmin resolution (about 10 km at equator), generated from national and subnational census data from various official sources. AEI is routinely reported by most countries in major international agriculture census database (e.g., FAOSTAT^30^, AQUASTAT^31^ and Eurostat^32^), whereas the area of land actually irrigated each year (AAI, also included in the GMIA) is reported less frequently, depending on the reporting country^20^. For data accuracy and consistency, we opted to use AEI over AAI for the irrigated area input.
- **Groundwater irrigation percentage**. The GMIA groundwater irrigation dataset also provides a global gridded estimate of the fraction of AEI using groundwater^2,21^. The data were derived from the consumptive water uses reported in national and subnational census and global crop water model outputs. The groundwater fraction estimates are for circa 2005 and given at a 5-arcmin resolution.
- **Irrigation water requirement**. The crop water use model, WATNEEDS^9^, is used to generate global maps of irrigation water requirements, termed blue water requirements (BWR). WATNEEDS assesses the vertical component of the soil water balance to obtain crop-specific monthly estimates of irrigation water requirements at 5-arcmin resolution. The model incorporates the 23 crop types, accounting for the majority of global crop water uses, and assumes a (stationary) distribution of crop types estimated for the year 2000^22^. The monthly irrigation water requirements are aggregated to annual BWRs and are summed over the 23 crops, accounting for multiple cropping, for reference years 2000, 2005, 2010, and 2015. To minimize errors caused by climate variability, the BWR estimates were averaged over a five-year period surrounding each reference year (e.g., 1998–2002 for the year 2000).
- **Cropland area**. The Global Land Analysis and Discovery (GLAD) cropland dataset^27^ contains global cropland (presence/absence) estimates at 30-m resolution for 2003, 2007, 2011, 2015, and 2019. Cropland includes land areas used for annual and perennial herbaceous crops for foods, forage, and biofuel, excluding perennial woody crops, permanent pastures, and shifting cultivation (slash and burn). The cropland images are generated from locally calibrated machine learning models with high-resolution remote sensing data performed in four-year intervals (2000-2003, 2004–2007, 2008–2011, 2012–2015, and 2016–2019) and named by the end year of the time period. We used the cropland extent image that covers the considered reference year (e.g., 2012–2015 for reference year 2015) for the irrigation water sources prediction.
- **Surface water features**. Surface water sources were identified using the HydroRIVERS dataset, part of the HydroSHEDS suite developed by the World Wildlife Fund (WWF)^33,34^. HydroRIVERS provides a comprehensive global vector database of river networks at high spatial resolution, derived from hydrologically conditioned elevation data and watershed boundaries. It includes river segments with consistent topology, flow direction, and stream order classification. For this study, we considered only major surface water features – specifically reservoirs, lakes, and rivers with a contributing drainage area greater than 25 km² – as potential sources of irrigation water. This filtering ensures the relevance of identified water bodies for agricultural use and reflects the assumption that small streams and minor tributaries are less likely to provide consistent irrigation supply at scale.
- **Urban area**. Urban areas were identified and excluded using the Global Human Settlement Layers (GHSL) dataset from the European Union Joint Research Center^35^. GHSL is a global, multi-temporal rural-urban classification dataset that integrates satellite imagery (Landsat and Sentinel-2), global population grids, census data, and volunteered geographic information. Global rural-urban classifications are available for 1975, 1990, 2000 and 2015 and follow the GHS-SMOD (Settlement Model), which implements the degree of urbanization (DEGURBA) framework at 1-km resolution. DEGURBA defines three classes based on population density and spatial contiguity: urban centers (high-density clusters), urban clusters (low-density clusters), and rural grid cells. In this study, we exclude all grid cells classified as urban centers to focus on non-urban irrigated areas.

### Preprocessing

**Fig. 2:**
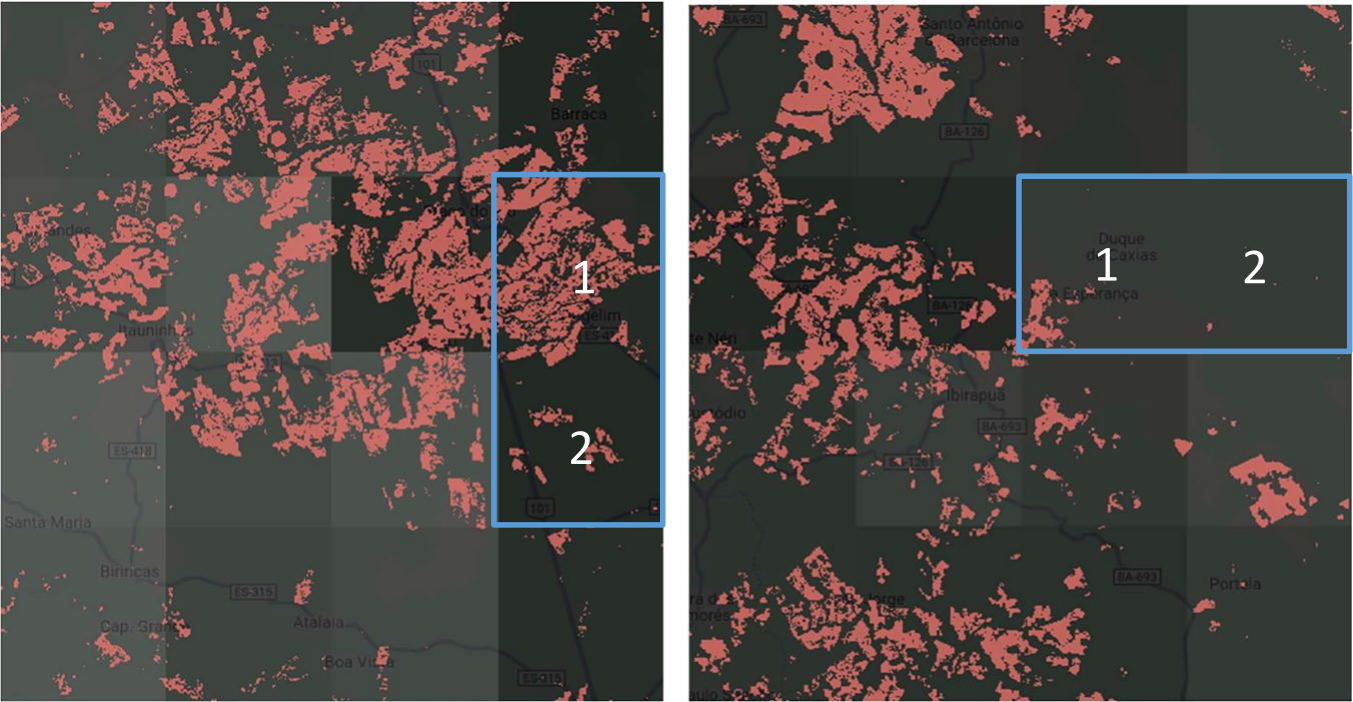
Examples of the discrepancy between AEI and cropland extent. The background displays the AEI grids at 10-km resolution, with the brighter areas representing higher AEI, while red indicates cropland areas at 30-m resolution. In both examples, AEI pixel 1 covers significantly more cropland than pixel 2 (highlighted by the rectangular blue boxes), yet the AEI percentage estimates (brightness) show no difference, indicating errors in the AEI estimation.

- **Resampling:** We reduced the spatial resolution of the GLAD cropland dataset from 30 meters to 60 meters to accommodate memory constraints imposed by the GEE platform. This upscaling was performed using majority (mode-based) resampling, where each new 60-meter pixel was classified as “cropland” if (i) at least 50% of the underlying 30-meter pixels were identified as cropland and (ii) it does not overlap with an identified urban pixel (see data sources). This method preserves dominant land cover patterns while reducing data volume for processing.
- **Harmonization:** Comparison between the GLAD cropland extent data and the GMIA AEI estimates revealed substantial discrepancies, largely due to the coarser resolution of the latter and its reliance on administrative unit-level statistics. For example, croplands may be concentrated in only part of an administrative unit (e.g., a county or district spanning multiple 10-km pixels), but AEI is assigned uniformly across all pixels within the unit, as illustrated in Fig. 2. This can lead to overestimates of AEI in areas with little or no cropland. To address this discrepancy between high-resolution cropland extent and coarser, administratively averaged AEI estimates, we implemented a harmonization procedure to improve the spatial consistency and plausibility of irrigation area estimates. The goal was to align AEI more closely with actual cropland distribution and biophysical irrigation needs, while minimizing errors introduced by administrative boundaries. We first converted AEI values to percentages relative to each 10-km grid cell, representing the share of the cell equipped for irrigation. We then applied a country-specific random forest^36^ model to adjust these AEI percentages based on biophysical estimates of irrigation water requirement (BWR; in $m/y$). Random forest^36^ is an ensemble learning method for classification and regression that builds a collection of decision trees and aggregates their predictions. Random forest algorithm is advantageous in reducing biases through bootstrap aggregation (bragging) and random feature selection and has been widely applied in remote sensing and hydrology fields^17,18,37,38^. In this study, we trained the random forest model to predict AEI percentage in 10-km cells using the BWR as input data. Training and predictions were performed independently for each country, under the assumption that farmers within a given country tend to follow similar irrigation practices. We set the maximum number of trees to 10 and minimum leaf to 1 to avoid overfitting and kept other parameters to the default values in GEE’s random forest implementation. The predicted AEI percentage accounts for variation in water demand across space and enhances comparability across countries. To ensure physical realism, we applied a feasibility check: if the predicted AEI percentage exceeded the cropland area percentage in a given grid cell (i.e., more than 100% of cropland irrigated), the value was capped at the cropland percentage. This constraint ensured that final AEI estimates remained consistent with observed cropland patterns and did not overestimate irrigated area.

### Irrigation water source assignment

The process assigns one of three water sources – rainfed, surface water, or groundwater – to each 60-meter cropland pixel, leveraging the spatial relationships between croplands and nearby surface water features. This assignment is based on several key assumptions. First, it is assumed that farmers will resort to groundwater irrigation when they either lack access to surface water –for example, due to the absence of necessary physical infrastructure – or when the cost of accessing surface water (including administrative and delivery fees) is higher than the cost of pumping groundwater; hence, distance to a surface water source is a key determinant of groundwater use for irrigation. Second, the percentage of irrigation sourced from groundwater per 5-arc minute pixel is assumed to not substantially vary from the groundwater irrigation percentage derived from the GMIA data (used here as the best available global estimate), even if the absolute area equipped for irrigation expands. This assumption enables the calculation of both surface water and groundwater irrigation areas within each 5-arc minute pixel and is validated using U.S. county-level water use data (see Usage Notes). Finally, spatial consolidation is considered a major feature of groundwater use. Irrigation infrastructure is often planned to serve multiple farms within an irrigation district to help justify the costs of construction and maintenance. Croplands located further away from surface water are less likely to have surface water access through irrigation infrastructure and, therefore, famers are more likely to install groundwater wells and use groundwater for irrigation. These wells are assumed to be situated centrally within contiguous irrigated cropland areas that are not supplied by surface water, in order to efficiently serve surrounding fields while minimizing conveyance losses.

Based on these assumptions, each 60-meter cropland pixel is assigned to surface water or groundwater irrigation until the total area allocated to each water source within the corresponding 5 arc-minute (approximately 10 km) GMIA grid cell is reached. The process begins by ranking all cropland pixels according to their distance to the nearest relevant surface water feature from the HydroRIVERS dataset (see data sources). Surface water irrigation is then assigned sequentially, starting with the pixel closest to a surface water source and continuing outward until the target surface water irrigation area is reached, defined as the total AEI multiplied by the complement of the groundwater irrigation fraction^2^ (Eq. 1).

1 $$SW=AEI\ast (1-GW)$$

where *SW* is the targeted surface water area, *AEI* denotes the total AEI area, and *GW* represents the groundwater irrigation fraction of the AEI area within a pixel cell.

Next, the remaining unassigned cropland pixels are ranked by their distance to the nearest non-cropland or surface-water-irrigated pixel, effectively capturing their distance to the edge of the remaining cropland patches. Groundwater irrigation is assigned starting with pixels farthest from the patch edges, moving outward until the groundwater AEI target is reached or all cropland pixels are assigned. Any remaining cropland pixels that have not been assigned to either surface water or groundwater irrigation are assumed to be rainfed.

### Uncertainty characterization

Even if we assume that our underlying assumptions are valid, two inherent sources of uncertainty still emerge from the downscaling process itself. The first source stems from the discrete nature of the resampled cropland dataset: each 60-m pixel is designated as cropland even if up to 50% of it is non-cropped. Consequently, each 60-m pixel is entirely assigned to one irrigation water source category (groundwater, surface water, or rainfed), which introduces noise into the results. We estimate this noise by upscaling our output to the native resolution of the GMIA dataset and comparing the predicted AEI, and its breakdown into surface water and groundwater components, with the GMIA values. Discrepancies, expressed as percentage point differences in the relative coverage for each 5-arc minute pixel, (PctPtErrorGWDownscale, PctPtErrorSWDownscale and PctPtErrorDownscale in Table 1) serve as an estimate of this uncertainty.

**Table 1:** Band descriptions of the irrigation water source images.

ID	Name	Description	Data Type	Range
0	cropClass	Five classes that indicate irrigation water sources and urban area. 0: non-cropland non-urban, 1: rainfed irrigation, 2: surface water irrigation, 3: groundwater irrigation, and 4: urban area.	Integer	[0,4]
1	PctPtErrorAEI	The error of overestimation in percentage point coverage of each 10-km pixel cell where AEIPct (band 7) exceeds cropMean (band 5) (%)	float	[0, 75.6]
2	PctPtErrorSWDownscale	Surface water irrigation error expressed in percentage point coverage of each 10-km pixel cell) compared to the targeted surface water irrigation computed using Eq. 1. (%)	float	[−1.44, 1.46]
3	PctPtErrorGWDownscale	The groundwater irrigation error (in percentage point coverage of each 10-km pixel cell) compared to GMIA’s groundwater irrigation percentage^2^ (%)	float	[−7.12, 18.89]
4	PctPtErrorDownscale	AEI prediction error (in percentage point coverage of each 10-km pixel cell) compared to AEI corrected with cropland data (AEI prediction - GMIA AEI^5^ adjusted by cropland area) (%)	float	[−7.055, 18.82]
5	cropMean	Cropland area per 10-km pixel cell upscaled from the GLAD dataset^27^ (m^2^)	float	[0, 8*1E7]
6	GWIrrFraction	The fraction of groundwater irrigation obtained from the GMIA dataset^2^ (-)	float	[0, 1]
7	AEIPct	The fraction of AEI area to the area of a 10-km pixel cell. (-)	float	[0, 1]

The second source of uncertainty arises from discrepancies between the GMIA AEI estimates and the GLAD cropland data that were not resolved in the preprocessing step. Specifically, in some GMIA grid cells, all GLAD cropland pixels may be classified as either surface water or groundwater irrigation before the expected target AEI indicated by the GMIA data are reached. This discrepancy is recorded during the downscaling process and reported as a bias, expressed as the percentage point difference between the AEI estimated by GMIA and the total cropland derived from GLAD (PctPtErrorAEI in Table 1). This bias estimate indicates regions where our dataset underestimates irrigated areas obtained from GMIA.

## Data Records

Data described in this paper are made available under a CC-BY 4.0 Creative Commons license and can be found in the Global Irrigation Water Sources Maps repository on figshare^39^ and GEE (https://code.earthengine.google.com/58dd5cf536982587f924e8a01777a448). This dataset contains four images of 2000, 2005, 2010, and 2015 at 60-m resolution. Each image has eight bands - Band 0 (cropClass) uses integer coding to indicate irrigation water sources and urban area, and bands 1 to 4 are the bias and uncertainty estimates described above. Bands 5–7 are relevant input data. The detailed descriptions of the bands, including units, are presented in Table 1.

The complete data‐accuracy assessment results described in the Technical Validation section are provided as Excel spreadsheet (.xlsx) in the Data Accuracy Assessment repository on figshare^40^. A separate Word document provides the column descriptions, units and data types. All files are released under a CC-BY 4.0 licence. The repository contains the following files:

- Table S1 Irrigation area prediction error estimate across U.S. states.xlsx
- Table S2 AEI prediction accuracy against Landsat-based Irrigation Dataset (LANID) masked by cropland area across U.S. states.xlsx
- Table S3 GMIA prediction accuracy against LANID masked by cropland across U.S. states.xlsx
- Table S4 GMIA-C prediction accuracy against LANID masked y cropland area across U.S. states.xlsx
- Table S5 Groundwater prediction accuracy against USGWD masked by cropland area
- across U.S. States.xlsx
- Table S6 Groundwater irrigation prediction accuracy against the Indian farmers survey
- by province.xlsx
- Data Dictionary Document.docx – column descriptions, units and data types of the tables.

## Technical Validation

Finding ground truth data has proven to be one of the most challenging tasks in validating the global irrigation water source dataset. With our best efforts, we collected three national and regional datasets from the literature and public websites and applied them to validate the downscaling results for the year 2015, which matched the epoch of the available data. The datasets applied for validation are the Landsat-based Irrigation Dataset (LANID)^17^, the United States Groundwater Well Database (USGWD) database^29^, and the Indian rice farmer survey^28^ (accessed through the International Maize and Wheat Improvement Center (CIMMYT) website: https://data.cimmyt.org/dataset.xhtml?persistentId=hdl:11529/10548656). Although we do not validate the downscaling results of 2000, 2005 and 2010, we expect similar accuracy because the inputs and methodology are consistent across years.

We carried out three distinct analyses. First, we compared our downscaled AEI (including surface and groundwater) to LANID, the irrigated cropland maps derived from remotely sensed data in the U.S.^17^. Second, we validated our groundwater irrigated cropland product against point location of 1.5 million wells in the U.S.^29^. Third, we validated our groundwater irrigated cropland data against surveyed locations of 8,355 irrigated fields in India^41^. The validation procedures and results, along with their relevant caveats associated with each analysis are presented in the following sections.

### Validation of downscaled AEI against remote-sensing derived products

To validate the downscaled AEI prediction, we first generated a new image consisting solely of surface water and groundwater irrigation pixels – referred to as the AEI prediction. This image was then compared with the 2015 LANID image^17^. The LANID dataset provides high-resolution (30-meter) maps of irrigation distribution, frequency, and changes across the contiguous U.S. from 1997 to 2017. Derived from Landsat imagery, LANID captures annual irrigation extent and intensity by integrating spectral indices, temporal composites, and machine-learning classification techniques. The dataset includes spatially explicit information on irrigated and non-irrigated croplands, allowing for the analysis of irrigation expansion, contraction, and temporal trends over two decades. As summarized in Table S1 (Data Accuracy Assessment repository^40^), the results show close agreement (underestimation by −4%) between our AEI prediction and the LANID dataset at the national level and a substantial improvement compared to the GMIA dataset, which overestimates LANID by +23%.

At the state level, the AEI prediction tends to overestimate irrigation areas in wetter states (states with average annual precipitation greater than 50 inches or 1,276 mm, which cover most south and eastern states in the U.S.) and underestimate them in arid and semi-arid states (states with average annual precipitation less than 30 inches or 762 mm including most of the mountain states, such as Arizona, Nevada, and New Mexico). Similar patterns are also observed when comparing LANID to the original GMIA dataset, though with enhanced magnitudes in the discrepancies. These biases may stem from the use of country-scale regression models for bias correction during our pre-processing step, which might underrepresent substantial regional heterogeneities in such a large country as the US, where persistent differences in crop water demand exist between the Eastern and Western parts of the country. Additionally, our approach considers a “typical” year by averaging multi-year data for BWR and cropland area. However, this assumption may not hold for 2015, which appears to have been an anomalously wet year: precipitation averaged across the contiguous U.S. was significantly higher than the 20^th^-century average^42^. To address these regional biases in future studies, a potential solution is to use data from a specific year and develop regression models tailored to climate zones within the study area. This approach could improve prediction accuracy by better capturing local climatic variability.

**Fig. 3:**
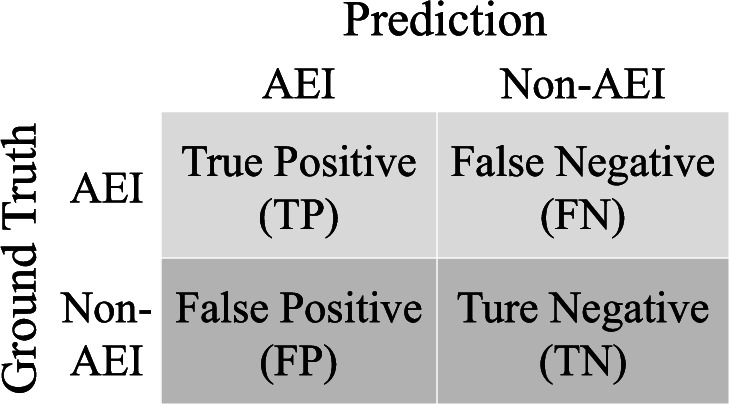
A 2-by-2 Confusion Matrix for binary classification of AEI (true) and non-AEI (false).

To assess prediction performance, we generated confusion matrices that tabulate the counts of predicted versus reference occurrences of AEI pixels, as illustrated in Fig. 3. From the true positive (TP), true negative (TN), false positive (FP) and false negative (FN) counted in these matrices, we then computed three accuracy metrics: producer accuracy, user accuracy, and overall accuracy as well as the F1 score. Producer accuracy, calculated as TP / (TP + FN), indicates the frequency with which the model correctly predicts AEI. User accuracy, computed as TP / (TP + FP), measures the likelihood that a location predicted to be equipped with irrigation is indeed equipped with irrigation. Overall accuracy is determined by (TP + TN) / (TP + TN + FP + FN), representing the rate at which both AEI and non-AEI are correctly predicted. Finally, F1 score is the harmonic mean of the user and producer accuracies (${F}_{1}=\frac{2{TP}}{2{TP}+{FP}+{FN}}$) and an indicator of class imbalance. The F1 score tends to align more closely with the lower of the user or producer accuracy values. All accuracy metrics are bounded between 0 and 1, as both numerator and denominator are non-negative by construction, with the denominator always greater than or equal to the numerator. To avoid division-by-zero errors, we define the metric as zero whenever both numerator and denominator are zero.

The accuracy assessment was conducted under two scenarios (unmasked and masked by cropland area) with three predictors: our AEI prediction, denoted as Pred, and two downscaled version of the GMIA datasets, denoted as GMIA and GMIA-C. The first downscaled GMIA version (labelled GMIA) employed a downscaling method in which 60-m pixels within each 10-km cell were randomly assigned to AEI with a probability equal to the AEI fraction of that 10-km cell. The second version (GMIA-C) refined this method by allocating AEI area only to 60-meter pixels within cropland areas, using cropland as an additional constraint. Masking by croplands enables a more meaningful comparison, as croplands cover only about 20% of the total land area in the U.S. Without this constraint, accuracy metrics may be skewed by an abundance of true negatives in non-cropland regions, potentially diminishing the ability to detect differences in the predictive accuracy of the datasets. The assessment results, summarized in Table 2, show that using the unmasked LANID dataset as the reference yields a high overall accuracy of 0.97 for our AEI prediction (Pred), with both producer and user accuracies above 0.5. While GMIA achieves a similar overall accuracy of 0.96 under the same condition, its producer and user accuracies are significantly lower (at around 0.3), highlighting its limitations in pixel-level precision.

**Table 2:** Summary of prediction accuracy comparison between the AEI prediction and two versions of downscaled GMIA datasets.

Reference	Cropland Mask	Predictor	Producer	User	Overall	F1score
LANID	Unmasked	Pred	0.50	0.58	0.97	0.54
	Unmasked	GMIA	0.34	0.30	0.96	0.32
	Masked	Pred	0.55	0.59	0.88	0.57
	Masked	GMIA	0.33	0.52	0.86	0.40
	Masked	GMIA-C	0.53	0.48	0.85	0.50

When using the cropland-masked LANID dataset, which removes most non-AEI areas and provides a more balanced accuracy evaluation, the AEI prediction (Pred) shows slight improvements in both producer (0.59) and user (0.55) accuracies, although the overall accuracy drops to 0.88. In comparison, GMIA reaches a user accuracy of 0.52 – only slightly below Pred – but its producer accuracy remains low (0.33), nearly identical to the unmasked case (0.34). The GMIA-C dataset, which integrates cropland data, achieves notably higher user and producer accuracies (0.48 and 0.53, respectively) than the unmasked GMIA, yet still falls short of the AEI prediction (Pred), which outperforms both GMIA-based datasets across all accuracy metrics in the masked scenario.

When comparing accuracy metrics by state, the AEI prediction tends to perform less promising and out-performed by GMIA-C in areas where rain-fed irrigation is prevalent (e.g., Connecticut and New York). This might be attributed to crop choices rather than the proximity to surface water sources. The prediction accuracy summaries by state are presented in  Tables S2-S4 (Data Accuracy Assessment repository).

### Validation of groundwater irrigation against well locations in the U.S

The USGWD database^29^ collects data from publicly accessible websites and direct contacts with the state agencies, providing the most comprehensive repository of 14.2 million well records for the contiguous U.S. Although the data has been processed to remove potential errors, the data records still contain missing fields and inconsistent coordinates, resulting in variable data quality between states. For example, Ohio, Indiana, Pennsylvania, and Minnesota have more than 30% of the records missing coordinate information according to the USGWD data quality assessment^29^. Moreover, the well records can indicate one or more water uses (e.g., irrigation, industrial, and domestic) and inactive wells. To better assess our prediction results, we focus on active wells for irrigation. We exclude wells that are inactive, with missing coordinates, or not used for irrigation. Multi-purpose wells are included if irrigation is listed as one of the designated water uses. After cleaning the data, the total number of effective data records is 1,505,371.

**Fig. 4:**
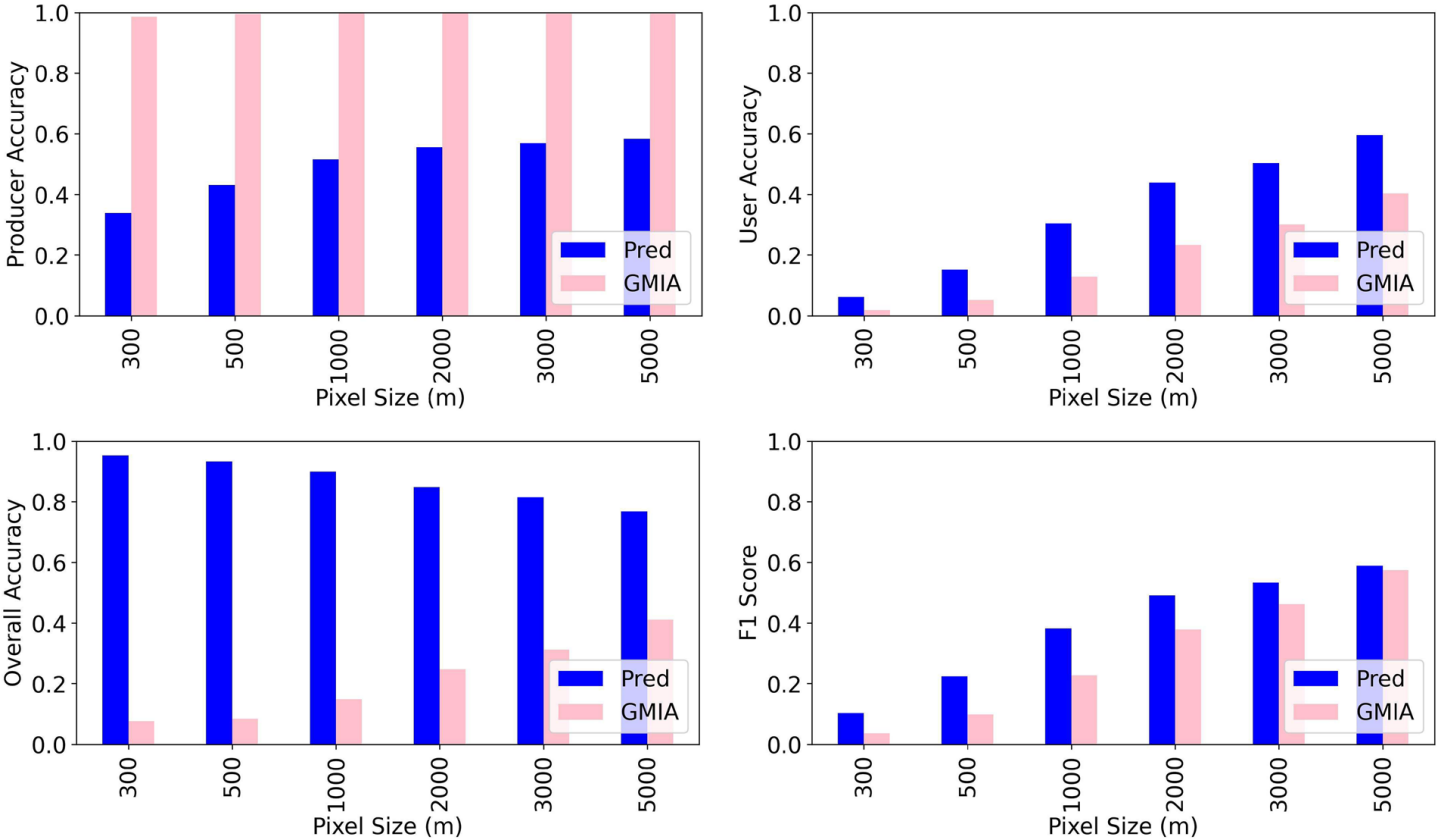
The groundwater irrigation prediction accuracies of the GMIA data and our downscaling method (labelled “Pred”) compared with USGWD data.

**Fig. 5:**
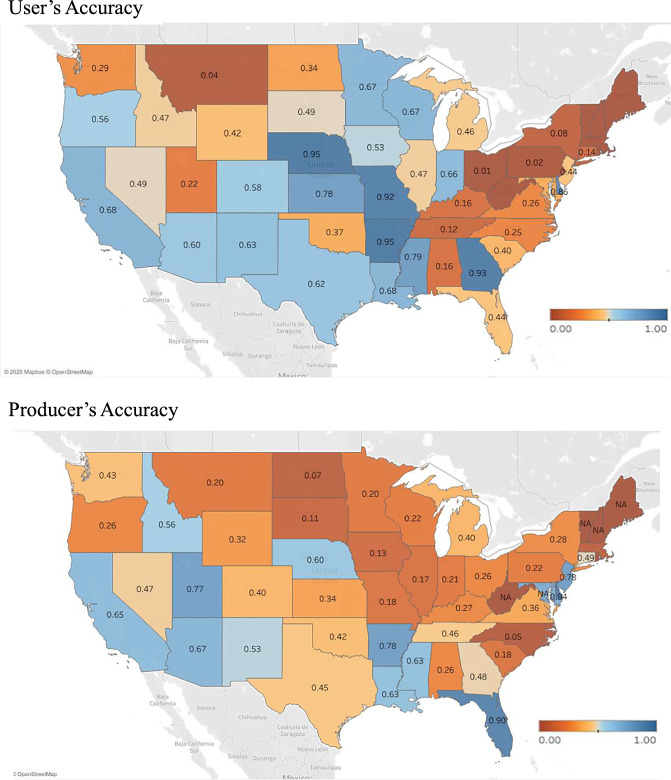
Validation results by State with USGWD data and 1-km pixel size.

The well records indicate the location of wells but does not map the fields using those wells for irrigation, which is what the datasets that we seek to validate (GMIA and AEI prediction) are predicting. We addressed this discrepancy by estimating the approximate area potentially irrigated by each well under a series of alternative assumptions. We assumed that any cropland location within a specified distance from a well (tested for 300 m, 500 m, 1 km, 2 km, 3 km, and 5 km) receives irrigation water from that well. For each assumed distance, we created a validation raster at the corresponding resolution using the USGWD well dataset: pixels overlapping one or more valid wells were assigned a value of 1 (indicating groundwater irrigation), and all other pixels were assigned a value of 0. The validation raster image was masked with cropland area to reduce excessive true negatives that may obscure prediction accuracy assessment. We then compared the validation raster with a rescaled version of our 60-m resolution groundwater irrigation dataset. To upscale to the relevant resolution, we applied a maximum-value aggregation method (i.e., a pixel is marked as groundwater-irrigated if any of its constituent high-resolution pixels are irrigated). The same scaling procedure was applied to the original GMIA groundwater irrigation data, which served as a benchmark. Figure 4 summarizes our key validation findings, with accuracies estimated at the national level and with the GMIA and our downscaled prediction labelled “GMIA” and “Pred,” respectively. Figure 5 provides state-level accuracy estimates for the “Pred” dataset.

At the national scale (Fig. 4), Pred achieves a user accuracy of 0.44 and an overall accuracy of 0.85 at the 2-km resolution, representing improvements of 0.20 and 0.60, respectively, over GMIA. These performance differences become even more telling when examining how accuracy varies with pixel size:

- GMIA begins as a 10-km layer that stores the area of groundwater-irrigated cropland. When rasterized for validation, any 10-km cell with non-zero area is converted to a full block of positive pixels. As a result, GMIA’s producer accuracy remains near-perfect across all evaluation scales because nearly every well falls within a positive cell. However, this also generates a high number of false positives, depressing user and overall accuracy. As the evaluation grid is coarsened, many of these false positives are absorbed into larger cells that now overlap at least one validation well. This artificially inflates the user and overall accuracies, and F1 scores from very poor (~0.1 at 300 m) to only marginally poor (~0.5 at 5 km). Despite the apparent improvement, the scaled GMIA map still substantially misrepresents irrigation.
- Pred, in contrast, originates as a fine-scale 60 m binary mask that downscales GMIA into plausible irrigated fields. When aggregated, many neighboring positives (for instance pixels belonging to a same field) are merged, improving alignment with the ground-truth raster. Producer accuracy rises modestly, while user accuracy improves substantially (from < 0.1 to ~0.6), and F1 follows the same trajectory, with both metrics always outperforming GMIA. Although Pred’s overall accuracy declines slightly as pixel size increases (from ~0.95 at 300 m to ~0.80 at 5 km), this drop reflects the expected loss of true negatives when small cropland pixels are merged into larger units. Even so, Pred retains a 20–30 percentage point lead in overall accuracy over GMIA at every resolution.

These results underline the value added by downscaling. By reallocating the 10-km GMIA totals to the most plausible 60-m locations, Pred transforms a coarse area-based product into a spatially explicit mask that dramatical reduces commission errors (higher user accuracy) while introducing a moderate amount of recall errors (lower producer accuracy). For comparison, we also tested random downscaling of the 10-km GMIA layer to finer resolutions. This alternative sharply reduced producer accuracy, as most wells no longer coincided with randomly placed irrigated pixels, reaffirming that the gains observed in Pred stem from informed spatial disaggregation, not resolution changes alone.

### Validation of groundwater irrigation location against field surveys in India

To complement the U.S. well‐infrastructure validation, we conducted a second analysis using survey data of rice farmers in India^41^. The survey data was collected by CIMMYT at site-level in 2018. The dataset contains field locations and interview responses from individual rice farmers in 49 districts within eight Indian states (Andhra Pradesh, Bihar, Chhattisgarth, Haryana, Odisha, Punjab, Uttar Pradesh, and West Bengal) to ensure broad agro-ecological coverage. The dataset has 8,355 entries, each recording the GPS coordinates of individual rice plots along with information on the sources of irrigation water. Unlike the US well dataset, which captures infrastructure locations, this dataset directly represents the location of irrigated fields, aligning more closely with the objective of our prediction. However, the data are not without limitations. Errors may arise from human entry mistakes or misreported coordinates – for example, locations being recorded at the respondent’s home instead of the actual rice plot. The irrigation water source field is also complex, listing a wide variety of sources (e.g., deep and shallow tube wells, ponds, lakes, rivers), and farmers are allowed to report multiple sources^43^.

For validation, we processed the survey data similarly to the USGWD analysis. Rice plots were assigned a value of 1 (groundwater irrigation) if any type of well was listed as the only or one of the irrigation sources, and 0 otherwise. The GPS data were converted to vector format and then to raster images at multiple resolutions (300 m, 500 m, 1 km, 2 km, 3 km, and 5 km). Non-cropland areas were masked, and our groundwater irrigation prediction was rescaled to each resolution using the maximum-value aggregation method. For each resolution, we performed pixel-to-pixel comparison and compute the validation metrics (overall accuracy, user accuracy, producer accuracy, and F1 score).

**Fig. 6:**
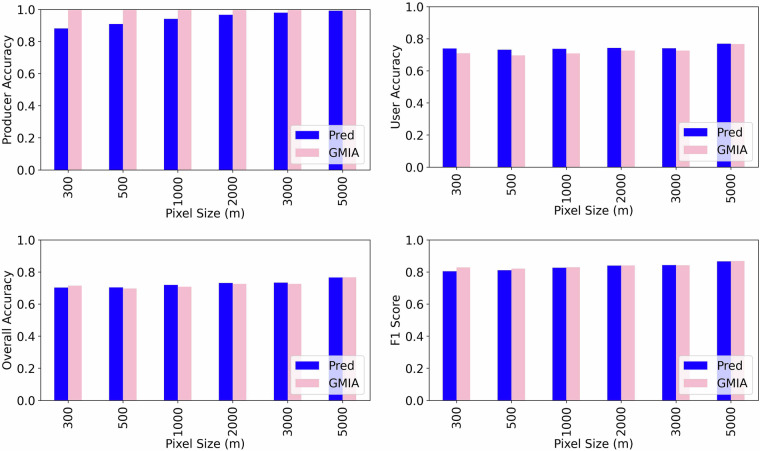
The groundwater irrigation prediction accuracy compared with Indian Farmers’ Survey data.

The validation results are summarized in Fig. 6. Both datasets, namely GMIA and our downscaled prediction (Pred), perform well across all spatial resolutions, with producer accuracy consistently above 0.85 and user and overall accuracies generally exceeding 0.70. However, unlike in the U.S., where downscaling yields substantial improvements in precision and overall accuracy, the benefits in India are more modest. This is primarily because groundwater irrigation is widespread in India, and most cropland pixels genuinely are irrigated. As a result, GMIA’s coarse 10-km grid, which marks entire cells as irrigated, aligns surprisingly well with the dense well infrastructure and the high prevalence of groundwater use. Its blanket classification captures nearly all true positives, and a large fraction of its potentially over-labelled pixels still contain irrigation, leading to relatively high (~0.70–0.75) user accuracy and overall accuracy even at fine resolutions. The Pred dataset improves upon this baseline, but only slightly: user accuracy rises a few points higher than GMIA’s at all, and overall accuracy is consistently a few percentage points higher. These gains reflect more precise targeting of actual irrigated plots and reduced commission error, especially at sub-kilometer resolutions. However, the small difference also highlights the diminishing marginal value of downscaling in highly irrigated, data-dense contexts where coarse data already performs reasonably well.

## Usage Notes

This dataset is a downscaled product of GMIA’s AEI rasters^5^, using information on crop water requirement, cropland distribution, groundwater irrigation percentage, and vicinity to surface water to determine the most likely main water source of each 60-meter cropland pixels: rainfed, surface water or groundwater irrigation. However, this simple classification overlooks an often ambiguous reality. Agriculture water users may choose to use different water sources based on precipitation, temperature, crop choices, access to water infrastructure (including wells, irrigation canal networks, and pumping stations), and other economic and regulatory factors. We encourage users to exercise caution when applying and interpreting the data, taking the limitations into account during their analyses and decision-making processes.

One caveat lies in the mixed uses of surface water and groundwater. This bias is embedded in the GMIA’s groundwater irrigation percentage data, derived from national and subnational statistics and propagates to this downscaled dataset. The conversion from water uses to irrigation area can create an illusion that a cropland receives water from only a single source. However, in reality, mixed uses of both water sources are not uncommon as arid and semiarid regions in which farmers tend to have intermittent and imbalanced surface water supply. Depending on weather conditions, seasons and crop rotation patterns, farmers may use alternative water sources. Our data represents average conditions over a five-year window (e.g., 2013–2017 for the 2015 dataset) and does not account for annual crop rotation. We therefore recommend interpreting the water source indicated by the downscaled dataset as the primary (rather than exclusive) source of crop water supply during the relevant period.

Second, an important assumption of the downscaling algorithm is that the percentage of groundwater-irrigated areas relative to AEI remains approximately constant within each 5 arcmin (10-km) grid cell. This assumption underpins the allocation of surface water and groundwater irrigation areas using the GMIA groundwater irrigation fraction. To test its validity, we used county-level water use data from the U.S. Geological Survey (USGS)^44^ for the Contiguous U.S., which includes detailed information on water use across categories, such as public supply, domestic, irrigation, thermoelectric power, industrial, and aquaculture, along with their respective water sources. We calculated the area-weighted average groundwater irrigation percentage for each county using U.S. county boundaries and compared these values with the GMIA groundwater irrigation percentage data, under the assumption that reported water uses are proportional to irrigated area. The resulting error metrics, with a mean squared error (MSE) of 4.75% and root-mean-squared error (RMSE) of 2.18%, suggest that the assumption of a relatively stable groundwater irrigation fraction is acceptable for the purpose of partitioning AEI into surface and groundwater components.

Third, a cropland’s access to surface water sources is assumed to be determined by its proximity to surface water features. The HydroRivers dataset^33^ used in this analysis is derived from a high-resolution (90-meter) digital elevation model (Shuttle Radar Topography Mission, SRTM)^45^. While this dataset can, in principle, detect rivers and surface water bodies, it fails to capture irrigation infrastructure, such as irrigation canal networks. Despite these limitations, we believe the core assumption – that surface water irrigation is more likely to occur near natural surface water bodies such as rivers or lakes – generally holds, given the high costs associated with conveying irrigation water over long distances.

Fourth, uncertainties in the validation process − both on the validation data themselves and on the spatial resolution at which validation is conducted − may affect the accuracy metrics reported. In the U.S., our validation draws on the USGWD well database, which, while comprehensive, includes known limitations such as outdated status information, missing infrastructure attributes, multipurpose water use classifications, and geolocation errors. Despite filtering to retain only active irrigation wells with valid coordinates, residual uncertainty remains. This is reflected in the state-level results (Fig. 5), where validation accuracy is substantially higher in states with completer and more precise well data (as indicated by the USGWD documentation), such as Arkansas, Delaware, Kansas, Mississippi, and Nebraska, where producer and user accuracies range between from 0.68 and 0.95.

A more fundamental challenge stems from the mismatch between the nature of the validation data and the spatial structure of the predictions. Our validation datasets in both the U.S. and India consist of discrete point data (well locations or surveyed fields), whereas the irrigation predictions are continuous area-based raster layers. To allow for comparison, both the predictions and the validation data needed to be aggregated to a common spatial resolution. This aggregation process introduces new tradeoffs that directly relate to the modifiable areal unit problem^46^ (MAUP), which refers to the statistical and interpretive biases that arise when spatial data are aggregated at arbitrary scales. As we show in both U.S. and India validations, accuracy metrics tend to increase with coarser aggregation (e.g., from 300 m to 5 km). This occurs because larger pixels smooth over spatial mismatch and field-level heterogeneity: a single irrigated sub-pixel can cause the entire cell to be marked as irrigated under maximum-value aggregation, converting many previous false negatives into true positives. However, this increase in apparent accuracy does not necessarily reflect improved model fidelity. In smallholder-dominated regions like India, aggregation masks meaningful variation in water source patterns. In sparsely irrigated regions such as parts of the U.S., it can inflate irrigated area by sweeping isolated irrigated pixels into larger false-positive zones. Therefore, we caution users against interpreting higher accuracy at coarser scales as a true gain in reliability. We recommend that users select a spatial resolution that aligns with the scale of the phenomenon under study (for example, ≤500 m in smallholder landscapes or 1–2 km in large-field systems) and interpret model performance in light of the MAUP challenge outlined above. Aggregation may improve alignment with noisy validation data and reduce random errors, but it also sacrifices spatial detail that is often critical for operational decision-making and local analyses.

To conclude, the dataset described in this paper is intended to support applications in hydrological modelling, agricultural water management, sustainability assessments, and the evaluation of groundwater depletion and surface water stress. The full downscaling pipeline is implemented in Google Earth Engine and made openly available, allowing users to replicate or modify the process. As new cropland, water use, or remote sensing data become available, the dataset can be efficiently updated to reflect more recent conditions. While subject to limitations related to mixed-source irrigation, resolution of input datasets, and assumptions around irrigation infrastructure access, this dataset represents a valuable resource for advancing global and regional analyses of irrigation dynamics.

## Supplementary information

Supplementary Tables S1-S6

## Data Availability

The codes for data processing, including downscaling, prediction and validation, are shared on Github: https://github.com/hfengwe1/GEE-GlobalGWIrr/tree/main.

## References

[1] Kahil, M. T., Dinar, A. & Albiac, J. Modeling water scarcity and droughts for policy adaptation to climate change in arid and semiarid regions. *J. Hydrol.* **522**, 95–109 (2015).

[2] Siebert, S. *et al*. Groundwater use for irrigation - A global inventory. *Hydrol. Earth Syst. Sci.* **14**, 1863–1880 (2010).

[3] Wada, Y., Wisser, D. & Bierkens, M. F. P. Global modeling of withdrawal, allocation and consumptive use of surface water and groundwater resources. *Earth Syst. Dyn.* **5**, 15–40 (2014).

[4] *The State of Food and Agriculture 2020. Overcoming Water Challenges in Agriculture*, 10.4060/cb1447en (FAO, Rome, 2020).

[5] Mehta, P. *et al*. Half of twenty-first century global irrigation expansion has been in water-stressed regions. *Nat. Water* **2**, 254–261 (2024).

[6] Voss, K. A. *et al*. Groundwater depletion in the Middle East from GRACE with implications for transboundary water management in the Tigris-Euphrates-Western Iran region. *Water Resour. Res.* **49**, 904–914 (2013). 23658469 10.1002/wrcr.20078 PMC3644870

[7] Castle, S. L. *et al*. Groundwater depletion during drought threatens future water security of the Colorado River Basin. *Geophys. Res. Lett.* **41**, 5904–5911 (2014). 25821273 10.1002/2014GL061055 PMC4373164

[8] Rodell, M., Velicogna, I. & Famiglietti, J. S. Satellite-based estimates of groundwater depletion in India. *Nature* **460**, 999–1002 (2009). 19675570 10.1038/nature08238

[9] Chiarelli, D. D. *et al*. The green and blue crop water requirement WATNEEDS model and its global gridded outputs. *Sci. Data* **7**, 1–9 (2020). 32811838 10.1038/s41597-020-00612-0 PMC7434899

[10] Khorrami, M. & Malekmohammadi, B. Effects of excessive water extraction on groundwater ecosystem services: Vulnerability assessments using biophysical approaches. *Sci. Total Environ.* **799**, 149304 (2021). 34375873 10.1016/j.scitotenv.2021.149304

[11] Crook, D. A. *et al*. Human effects on ecological connectivity in aquatic ecosystems: Integrating scientific approaches to support management and mitigation. *Sci. Total Environ.* **534**, 52–64 (2015). 25917446 10.1016/j.scitotenv.2015.04.034

[12] Rohde, M. M. *et al*. Groundwater-dependent ecosystem map exposes global dryland protection needs. *Nature* **632**, 101–107 (2024). 39020182 10.1038/s41586-024-07702-8 PMC11291274

[13] Mullen, C. *et al*. Hydro Economic Asymmetries and Common-Pool Overdraft in Transboundary Aquifers. *Water Resour. Res*. **58** (2022).

[14] Lowder, S. K., Skoet, J. & Raney, T. The Number, Size, and Distribution of Farms, Smallholder Farms, and Family Farms Worldwide. *World Dev.* **87**, 16–29 (2016).

[15] Döll, P. & Siebert, S. Global modeling of irrigation water requirements. *Water Resour. Res.* **38**, 8-1–8–10 (2002).

[16] Ambika, A. K., Wardlow, B. & Mishra, V. Remotely sensed high resolution irrigated area mapping in India for 2000 to 2015. *Sci. Data* **3**, 1–14 (2016). 10.1038/sdata.2016.118 PMC5170598 27996974

[17] Xie, Y., Gibbs, H. K. & Lark, T. J. Landsat-based Irrigation Dataset (LANID): 30 m resolution maps of irrigation distribution, frequency, and change for the US, 1997-2017. *Earth Syst. Sci. Data* **13**, 5689–5710 (2021).

[18] Zhang, C., Dong, J. & Ge, Q. Mapping 20 years of irrigated croplands in China using MODIS and statistics and existing irrigation products. *Sci. Data* **9**, 1–12 (2022). 35840621 10.1038/s41597-022-01522-z PMC9287319

[19] Siebert, S. *et al*. Hydrology and Earth System Sciences Development and validation of the global map of irrigation areas. *Hydrol. Earth Syst. Sci.* **9**, 535–547 (2005).

[20] Siebert, S. *et al*. A global data set of the extent of irrigated land from 1900 to 2005. *Hydrol. Earth Syst. Sci.* **19**, 1521–1545 (2015).

[21] Siebert, S. *et al*. *Update of the Digital Global Map of Irrigation Areas to Version 5*. *Crop Sci. Resour. Conserv. Rheinische Friedrich-Wilhelms-Univ. Bonn Ger*, 10.13140/2.1.2660.6728 (2013).

[22] Portmann, F. T., Siebert, S. & Döll, P. MIRCA2000—Global monthly irrigated and rainfed crop areas around the year 2000: A new high‐resolution data set for agricultural and hydrological modeling. *Global Biogeochem. Cycles* **24** (2010).

[23] Nagaraj, D. *et al*. Advances in Water Resources A new dataset of global irrigation areas from 2001 to 2015. *Adv. Water Resour.* **152**, 103910 (2021).

[24] Meier, J., Zabel, F. & Mauser, W. A global approach to estimate irrigated areas – a comparison between different data and statistics. 1119–1133 (2018).

[25] Aeschbach-Hertig, W. & Gleeson, T. Regional strategies for the accelerating global problem of groundwater depletion. *Nat. Geosci.* **5**, 853–861 (2012).

[26] Richey, A. S. *et al*. Quantifying renewable groundwater stress with GRACE. *Water Resour. Res.* **51**, 5217–5237 (2015). 26900185 10.1002/2015WR017349 PMC4744761

[27] Potapov, P. *et al*. Global maps of cropland extent and change show accelerated cropland expansion in the twenty-first century. *Nat. Food* **3**, 19–28 (2022). 37118483 10.1038/s43016-021-00429-z

[28] Ajay, A. *et al*. Large survey dataset of rice production practices applied by farmers on their largest farm plot during 2018 in India. *Data Br.* **45**, 108625 (2022). 10.1016/j.dib.2022.108625 PMC9679526 36426044

[29] Lin, C. Y. *et al*. A database of groundwater wells in the United States. *Sci. Data* **11**, 1–14 (2024). 38575591 10.1038/s41597-024-03186-3 PMC10995170

[30] FAO. FAOSTAT. https://www.fao.org/faostat/en/#home.

[31] FAO. AQUASTAT. https://www.fao.org/aquastat/en/.

[32] European Union. Eurostat. https://ec.europa.eu/eurostat.

[33] Lehner, B. & Grill, G. Global river hydrography and network routing: Baseline data and new approaches to study the world’s large river systems. *Hydrol. Process.* **27**, 2171–2186 (2013).

[34] Lehner, B., Verdin, K. & Jarvis, A. New global hydrography derived from spaceborne elevation data. *Eos (Washington. DC).* **89**, 93–94 (2008).

[35] Joint Research Centre. *GHSL Data Package 2023*, 10.2760/098587 (2023).

[36] Breiman, L. Random forests. *in Machine Learning* **45**, 5–32 (2001).

[37] Ketchum, D. *et al*. IrrMapper: A machine learning approach for high resolution mapping of irrigated agriculture across the Western U.S. *Remote Sens.* **12**, 1–23 (2020).

[38] Stein, L. *et al*. A. How Do Climate and Catchment Attributes Influence Flood Generating Processes? A Large-Sample Study for 671 Catchments Across the Contiguous USA. *Water Resour. Res.* **57**, 1–21 (2021).

[39] Hung, F. *et al*., Global Irrigation Water Sources Maps. *figshare*, 10.6084/m9.figshare.c.7318916 (2024).

[40] Hung, F. *et al*. Data Accuracy Assessment. *figshare*10.6084/m9.figshare.29621924 (2024).

[41] Ajay, A. *et al*. Large-scale data of crop production practices applied by farmers on their largest rice plot during 2018 in eight Indian states. *CIMMYT Research Data & Software Repository Network* https://data.cimmyt.org/dataset.xhtml?persistentId=hdl:11529/10548656 (2022).

[42] NOAA National Centers for Environmental Information. *Monthly National Climate Report for Annual 2015*. https://www.ncei.noaa.gov/access/monitoring/monthly-report/national/201513 (2016).

[43] Ajay, A. *et al*. Large-scale data of crop production practices applied by farmers on their largest rice plot during 2018 in eight Indian states - LDS Codebook. *CIMMYT Res. Data Softw. Repos. Netw*. **V3** (2018).

[44] U.S. Geological Survey. *Estimated Use of Water in the United States County-Level Data for 2015*. https://www.sciencebase.gov/catalog/item/get/5af3311be4b0da30c1b245d8, 10.5066/F7TB15V5 (2018).

[45] US Geological Survey. USGS EROS Archive - Digital Elevation - Shuttle Radar Topography Mission (SRTM) 1 Arc-Second Global. https://www.usgs.gov/centers/eros/science/usgs-eros-archive-digital-elevation-shuttle-radar-topography-mission-srtm-1?qt-science_center_objects=0#qt-science_center_objects, 10.5066/F7PR7TFT (2018).

[46] Openshaw, S. *The Modifiable Areal Unit Problem: Concepts and Techniques in Modern Geography*. GeoBooks, Norwich, UK (1984).

