# Supplementary material for: Downscaled global 60-meter resolution estimates of irrigation water sources (2000–2015)
Source: Sci Data. 2025 Oct 9;12:1632. doi: 10.1038/s41597-025-05920-x (PMC12511346; doi:10.1038/s41597-025-05920-x)
Supplement: Supplementary file 1 — Supplementary Tables S1-S6 [file 41597_2025_5920_MOESM1_ESM.pdf]

Supplementary Tables for Manuscript "Downscaled global 60-meter resolution estimates of irrigation water sources (2000-2015)"

Authors: Fengwei Hung, Davide Danilo Chiarelli[3], James S. Famiglietti[2] , Marc F. Müller

Date: August 2025

Data Dictionary

This document describes the columns for each of the supplementary tables (S1–S6). For each table, the following fields are provided:

- **Column Name**: The exact name of the column in the dataset.
- **Description**: Detailed explanation of the column's contents.
- **Data Type**: Type of data stored (e.g., Integer, Float, String).
- **Units**: Measurement units, if applicable.
- **Notes**: Any additional information or coding conventions.

Table S1. Irrigation Area Prediction Error Estimate Across US States

Column Name	Description	Data Type	Units	Notes
State	US state name	String	—	
AEI Predict	This study's AEI prediction	Float	km^2	
GMIA	GMIA's AEI prediction	Float	km^2	Mehta et al. (2024)[1]
LANID	Reference AEI area (ground truth)	Float	km^2	Xie et al. (2021)[2]
AEI Predict (Err)	Percentage error of our AEI downscaled data relative to reference	Float	$\%^2$	= (AEI Predict - LANID)/ LANID) × 100%
GMIA (Err)	Percentage error of the GMIA data relative to reference	Float	%	= (error absolute / AEI reference) × 100

Table S2. AEI Prediction Accuracy Against LANID Masked by Cropland Area Across US States

Column Name	Description	Data Type	Units	Notes

State	Two-letter US state abbreviation	String	—	
Producer	The proportion of actual AEI pixels (indicated by LANID) that our model correctly predicts as AEI.	Float	—	TP / (TP + FN)
User	The proportion of pixels predicted as AEI that are actually AEI.	Float	—	TP / (TP + FP)
NonAEI Producer	The proportion of actual non-AEI pixels (i.e., areas not equipped with irrigation; indicated by LANID) that our model correctly predicts as non-AEI.	Float	—	
NonAEI User	The proportion of pixels predicted as non-AEI that are actually non-AEI.	Float	—	
Overall Accuracy	Percentage of correctly classified AEI pixels	Float	—	(TP + TN) / (TP + TN + FP + FN)
F1 score	Harmonic mean of precision and recall	Float	—	$F_1 = \frac{2TP}{2TP + FP + FN}$

Table S3 and S4. Columns identical to Table S2 except the predictors are GMIA' AEI randomly downscaled (GMIA) and GMIA's AEI randomly allocated to 60-m cropland area pixels (GMIA-C), respectively.

Table S5. Groundwater prediction accuracy against USGWD masked by cropland area across US States

This dictionary describes the naming convention and details of columns in the accuracy tables for groundwater irrigation predictions. Column names follow the pattern: **`A-B####`**

- **A**: Data source/model
  - **G**: GMIA (Global Map of Irrigation Areas) benchmark
  - **P**: Our prediction model
- **B**: Accuracy metric
  - **P**: Producer accuracy (true positive rate for groundwater irrigation)
  - **U**: User accuracy (positive predictive value for groundwater irrigation)
  - **NP**: Producer accuracy for non-groundwater irrigated pixels (true negative rate)

  - `NU`: User accuracy for non-groundwater irrigated pixels (negative predictive value)
  - `ALLGW`: Overall accuracy (percentage of all pixels correctly classified)
  - `F1score`: F1 score (harmonic mean of precision and recall)
- `####`: Spatial resolution (`300`, `500`, `1000`, `2000`, `5000`) of the aggregated grid (in meters).

Column Name	Description	Data Type	Units	Notes
State	Two-letter US state abbreviation	Sring	—	
G-P300	GMIA producer accuracy at 300 m resolution (true positive rate)	Float	—	
G-U300	GMIA user accuracy at 300 m resolution (positive predictive value)	Float	—	
G-NP300	GMIA non-groundwater producer accuracy at 300 m resolution (true negative rate)	Float	—	
G-NU300	GMIA non-groundwater user accuracy at 300 m resolution (negative predictive value)	Float	—	
G-ALLGW300	GMIA overall accuracy at 300 m resolution	Float	—	
G-F1score300	GMIA F1 score at 300 m resolution	Float	—	
P-P300	Prediction model producer accuracy at 300 m resolution	Float	—	
P-U300	Prediction model user accuracy at 300 m resolution	Float	—	
P-NP300	Prediction model non-groundwater producer accuracy at 300 m resolution	Float	—	
P-NU300	Prediction model non-groundwater user accuracy at 300 m resolution	Float	—	
P-ALLGW300	Prediction model overall accuracy at 300 m resolution	Float	—	
P-F1score300	Prediction model F1 score at 300 m resolution	Float	—	

- Although the data dictionaries of other resolutions are omitted for brevity, they could be easily generated by replacing the number in the column names and description.

Table S6. Groundwater irrigation prediction accuracy against the Indian farmers survey by province

The column naming follows the same pattern as explained in Table S5 (**`A-B####)`**. The only difference is the column "State" is replaced by "Province" that stores the Indian province names.

Table S1 Irrigation area prediction error estimate across U.S. states.xlsx

State	AEI Predict	GMIA	LANID	AEI Predict (Er	GMIA (Err)
Alabama	500	593	798	-37%	-26%
Arizona	3072	3675	3356	-8%	10%
Arkansas	19587	23963	19046	3%	26%
California	23197	29188	30349	-24%	-4%
Colorado	9495	12298	9796	-3%	26%
Connecticut	36	49	10	276%	405%
Delaware	617	794	661	-7%	20%
District of Columbi	0	0	0	na	na
Florida	4042	4583	4730	-15%	-3%
Georgia	5918	6977	4443	33%	57%
Idaho	12317	16914	13294	-7%	27%
Illinois	2700	3546	4390	-38%	-19%
Indiana	2067	2732	2742	-25%	0%
Iowa	887	1194	1681	-47%	-29%
Kansas	14241	18159	13000	10%	40%
Kentucky	232	292	398	-42%	-27%
Louisiana	5415	6373	4510	20%	41%
Maine	46	67	117	-61%	-43%
Maryland	521	669	654	-20%	2%
Massachusetts	72	98	25	189%	291%
Michigan	2583	3513	3198	-19%	10%
Minnesota	2503	3585	2842	-12%	26%
Mississippi	6929	8352	5849	18%	43%
Missouri	6099	7661	7143	-15%	7%
Montana	6633	9731	6917	-4%	41%
Nebraska	39137	52069	39278	0%	33%
Nevada	1685	2209	2320	-27%	-5%
New Hampshire	3	4	10	-72%	-61%
New Jersey	402	524	293	37%	79%
New Mexico	2847	3454	2718	5%	27%
New York	231	313	232	-1%	35%
North Carolina	1232	1517	615	100%	147%
North Dakota	1119	1644	2261	-51%	-27%
Ohio	160	211	217	-26%	-3%
Oklahoma	2470	3050	2619	-6%	16%
Oregon	5983	8369	6598	-9%	27%
Pennsylvania	100	131	48	109%	174%
Rhode Island	8	10	8	-1%	33%
South Carolina	566	682	766	-26%	-11%
South Dakota	2107	2932	3106	-32%	-6%
Tennessee	302	373	1064	-72%	-65%
Texas	25263	30282	19859	27%	52%
Utah	3528	4623	3879	-9%	19%
Vermont	3	4	10	-75%	-65%
Virginia	489	617	317	54%	95%

Washington	6827	9986	6734	1%	48%
West Virginia	5	7	4	21%	56%
Wisconsin	1978	2758	2195	-10%	26%
Wyoming	2698	3702	4094	-34%	-10%
**US**	**228852**	**294475**	**239192**	**-4%**	**23%**

Table S2 AEI prediction accuracy against Landsat-based Irrigation Dataset (LANID)

State	Producer	User	NonAEI Producer	NonAEI User	Overall	F1 score
AL	0.08	0.21	0.97	0.91	0.88	0.12
AR	0.77	0.79	0.62	0.59	0.72	0.78
AZ	0.8	0.89	0.45	0.3	0.75	0.84
CA	0.89	0.78	0.27	0.46	0.73	0.83
CO	0.5	0.5	0.82	0.83	0.74	0.5
CT	0.05	0.02	0.97	0.99	0.96	0.03
DC	-	-	1	1	1	-
DE	0.44	0.56	0.86	0.79	0.73	0.49
FL	0.73	0.64	0.66	0.75	0.69	0.68
GA	0.58	0.48	0.82	0.87	0.76	0.52
IA	0.1	0.22	1	0.99	0.98	0.14
ID	0.71	0.8	0.72	0.61	0.72	0.75
IL	0.14	0.26	0.98	0.96	0.95	0.18
IN	0.15	0.26	0.98	0.96	0.94	0.19
KS	0.29	0.27	0.91	0.91	0.84	0.28
KY	0.05	0.14	0.99	0.98	0.98	0.07
LA	0.51	0.43	0.78	0.83	0.71	0.47
MA	0.2	0.06	0.94	0.98	0.93	0.09
MD	0.24	0.41	0.97	0.93	0.9	0.3
ME	0.03	0.15	0.99	0.95	0.95	0.05
MI	0.25	0.42	0.97	0.93	0.91	0.31
MN	0.17	0.25	0.98	0.97	0.96	0.2
MO	0.47	0.62	0.96	0.93	0.9	0.53
MS	0.64	0.56	0.75	0.81	0.72	0.6
MT	0.38	0.39	0.93	0.93	0.87	0.38
NC	0.05	0.05	0.97	0.97	0.95	0.05
ND	0.02	0.06	0.99	0.98	0.97	0.03
NE	0.66	0.7	0.78	0.74	0.72	0.68
NH	0.02	0.2	1	0.98	0.98	0.03
NJ	0.3	0.26	0.89	0.91	0.82	0.28
NM	0.57	0.57	0.61	0.61	0.59	0.57
NV	0.65	0.86	0.52	0.24	0.63	0.74
NY	0.08	0.17	1	0.99	0.99	0.11
OH	0	0.01	1	1	1	0
OK	0.19	0.23	0.96	0.94	0.91	0.21
OR	0.49	0.57	0.77	0.71	0.66	0.53
PA	0.02	0.03	1	1	1	0.02
RI	0.11	0.17	0.96	0.94	0.91	0.14
SC	0.2	0.41	0.97	0.92	0.9	0.27
SD	0.09	0.16	0.98	0.96	0.95	0.11

TN	0.06	0.29	0.99	0.94	0.94	0.1
TX	0.51	0.42	0.8	0.85	0.74	0.46
UT	0.66	0.73	0.53	0.46	0.62	0.69
VA	0.09	0.11	0.99	0.98	0.97	0.1
VT	-	-	1	1	1	-
WA	0.44	0.45	0.82	0.81	0.72	0.44
WI	0.25	0.37	0.98	0.96	0.95	0.3
WV	-	-	1	1	1	-
WY	0.57	0.76	0.69	0.47	0.61	0.65
**US**	**0.55**	**0.59**	**0.94**	**0.93**	**0.88**	**0.57**

Table S3 GMIA prediction accuracy against LANID masked by cropland across U.S.

**State**	**Producer**	**User**	**onAEI Produ**	**NonAEI User**	**Overall**	**F1 score**
AL	0.03	0.16	0.98	0.91	0.9	0.05
AR	0.57	0.71	0.64	0.5	0.6	0.63
AZ	0.34	0.85	0.75	0.22	0.42	0.49
CA	0.64	0.76	0.51	0.36	0.6	0.69
CO	0.24	0.45	0.9	0.77	0.73	0.32
CT	0.03	0.03	0.99	0.99	0.98	0.03
DC	-	-	1	1	1	-
DE	0.15	0.35	0.87	0.69	0.65	0.21
FL	0.46	0.7	0.86	0.69	0.69	0.56
GA	0.16	0.37	0.91	0.76	0.72	0.22
IA	0.07	0.14	0.99	0.99	0.98	0.1
ID	0.46	0.73	0.75	0.49	0.58	0.57
IL	0.12	0.22	0.98	0.96	0.95	0.15
IN	0.08	0.13	0.97	0.96	0.93	0.1
KS	0.2	0.25	0.92	0.9	0.84	0.22
KY	0.03	0.05	0.99	0.98	0.97	0.04
LA	0.25	0.39	0.87	0.77	0.71	0.31
MA	0.03	0.06	0.99	0.98	0.97	0.04
MD	0.11	0.27	0.97	0.92	0.89	0.16
ME	0.02	0.1	0.99	0.96	0.95	0.03
MI	0.1	0.24	0.97	0.92	0.89	0.14
MN	0.08	0.13	0.98	0.97	0.96	0.1
MO	0.36	0.52	0.96	0.92	0.89	0.43
MS	0.4	0.48	0.79	0.74	0.67	0.44
MT	0.15	0.31	0.96	0.9	0.87	0.2
NC	0.02	0.04	0.99	0.97	0.96	0.03
ND	0.02	0.05	0.99	0.98	0.97	0.03
NE	0.42	0.59	0.78	0.63	0.62	0.49
NH	0	0.02	1	0.98	0.98	0.01
NJ	0.1	0.21	0.95	0.88	0.84	0.14
NM	0.16	0.54	0.88	0.54	0.54	0.24
NV	0.18	0.84	0.87	0.22	0.32	0.3
NY	0.03	0.06	0.99	0.99	0.99	0.04
OH	0.01	0.01	1	1	0.99	0.01
OK	0.09	0.2	0.97	0.93	0.91	0.12

OR	0.24	0.5	0.85	0.65	0.62	0.33
PA	0.01	0.01	1	1	0.99	0.01
RI	0.01	0.05	0.98	0.93	0.91	0.02
SC	0.05	0.19	0.98	0.9	0.88	0.08
SD	0.05	0.11	0.98	0.96	0.94	0.07
TN	0.06	0.14	0.98	0.94	0.92	0.08
TX	0.28	0.41	0.89	0.81	0.75	0.33
UT	0.27	0.67	0.77	0.39	0.46	0.39
VA	0.02	0.05	0.99	0.98	0.97	0.03
VT	0	0	0.99	1	0.99	0
WA	0.23	0.44	0.9	0.78	0.74	0.3
WI	0.09	0.21	0.98	0.96	0.94	0.13
WV	0.02	0.01	1	1	0.99	0.01
WY	0.19	0.72	0.88	0.4	0.45	0.3
**US**	**0.33**	**0.52**	**0.95**	**0.9**	**0.86**	**0.4**

Table S4 GMIA-C prediction accuracy against LANID masked y cropland area across US States

**State**	**Producer**	**User**	**onAEI Produ**	**NonAEI User**	**Overall**	**F1 score**
AL	0.03	0.16	0.98	0.91	0.9	0.05
AR	0.57	0.71	0.64	0.5	0.6	0.63
AZ	0.34	0.85	0.75	0.22	0.42	0.49
CA	0.64	0.76	0.51	0.36	0.6	0.69
CO	0.24	0.45	0.9	0.77	0.73	0.32
CT	0.03	0.03	0.99	0.99	0.98	0.03
DC	-	-	1	1	1	-
DE	0.15	0.35	0.87	0.69	0.65	0.21
FL	0.46	0.7	0.86	0.69	0.69	0.56
GA	0.16	0.37	0.91	0.76	0.72	0.22
IA	0.07	0.14	0.99	0.99	0.98	0.1
ID	0.46	0.73	0.75	0.49	0.58	0.57
IL	0.12	0.22	0.98	0.96	0.95	0.15
IN	0.08	0.13	0.97	0.96	0.93	0.1
KS	0.2	0.25	0.92	0.9	0.84	0.22
KY	0.03	0.05	0.99	0.98	0.97	0.04
LA	0.25	0.39	0.87	0.77	0.71	0.31
MA	0.03	0.06	0.99	0.98	0.97	0.04
MD	0.11	0.27	0.97	0.92	0.89	0.16
ME	0.02	0.1	0.99	0.96	0.95	0.03
MI	0.1	0.24	0.97	0.92	0.89	0.14
MN	0.08	0.13	0.98	0.97	0.96	0.1
MO	0.36	0.52	0.96	0.92	0.89	0.43
MS	0.4	0.48	0.79	0.74	0.67	0.44
MT	0.15	0.31	0.96	0.9	0.87	0.2
NC	0.02	0.04	0.99	0.97	0.96	0.03
ND	0.02	0.05	0.99	0.98	0.97	0.03
NE	0.42	0.59	0.78	0.63	0.62	0.49
NH	0	0.02	1	0.98	0.98	0.01
NJ	0.1	0.21	0.95	0.88	0.84	0.14

NM	0.16	0.54	0.88	0.54	0.54	0.24
NV	0.18	0.84	0.87	0.22	0.32	0.3
NY	0.03	0.06	0.99	0.99	0.99	0.04
OH	0.01	0.01	1	1	0.99	0.01
OK	0.09	0.2	0.97	0.93	0.91	0.12
OR	0.24	0.5	0.85	0.65	0.62	0.33
PA	0.01	0.01	1	1	0.99	0.01
RI	0.01	0.05	0.98	0.93	0.91	0.02
SC	0.05	0.19	0.98	0.9	0.88	0.08
SD	0.05	0.11	0.98	0.96	0.94	0.07
TN	0.06	0.14	0.98	0.94	0.92	0.08
TX	0.28	0.41	0.89	0.81	0.75	0.33
UT	0.27	0.67	0.77	0.39	0.46	0.39
VA	0.02	0.05	0.99	0.98	0.97	0.03
VT	0	0	0.99	1	0.99	0
WA	0.23	0.44	0.9	0.78	0.74	0.3
WI	0.09	0.21	0.98	0.96	0.94	0.13
WV	0.02	0.01	1	1	0.99	0.01
WY	0.19	0.72	0.88	0.4	0.45	0.3
**US**	**0.33**	**0.52**	**0.95**	**0.9**	**0.86**	**0.4**

Table S5 Groundwater prediction accuracy against USGWD masked by cropland area

State	G-P300	G-U300	G-NP300	G-NU300	G-AllGW300	-F1score30
AL	0.95	0.01	0.16	1.00	0.16	0.01
AR	1.00	0.12	0.02	1.00	0.13	0.21
AZ	0.99	0.04	0.02	0.97	0.06	0.08
CA	1.00	0.02	0.01	0.98	0.03	0.05
CO	0.99	0.03	0.01	0.98	0.04	0.06
CT	0.77	0.00	0.22	1.00	0.22	0.01
DC	0.00	0.00	0.50	1.00	0.50	0.00
DE	1.00	0.17	0.00	0.96	0.17	0.30
FL	0.99	0.16	0.03	0.92	0.18	0.28
GA	1.00	0.04	0.01	1.00	0.05	0.08
IA	1.00	0.00	0.01	1.00	0.01	0.00
ID	0.97	0.03	0.02	0.95	0.05	0.07
IL	1.00	0.00	0.00	0.99	0.00	0.01
IN	1.00	0.00	0.00	1.00	0.00	0.01
KS	1.00	0.02	0.00	1.00	0.02	0.03
KY	1.00	0.00	0.01	1.00	0.01	0.01
LA	1.00	0.04	0.01	0.99	0.05	0.08
MA	0.50	0.02	0.42	0.97	0.42	0.04
MD	0.99	0.05	0.02	0.98	0.07	0.09
ME	0.98	0.00	0.04	1.00	0.04	0.00
MI	1.00	0.01	0.00	0.99	0.01	0.02
MN	1.00	0.00	0.00	1.00	0.01	0.01
MO	1.00	0.01	0.02	1.00	0.03	0.01
MS	1.00	0.09	0.00	1.00	0.09	0.17
MT	0.42	0.00	0.51	1.00	0.51	0.00
NC	0.95	0.00	0.24	1.00	0.24	0.00

ND	1.00	0.00	0.09	1.00	0.09	0.00
NE	1.00	0.06	0.00	0.99	0.07	0.12
NH	0.84	0.00	0.20	1.00	0.20	0.01
NJ	0.99	0.09	0.01	0.93	0.10	0.16
NM	1.00	0.09	0.02	0.99	0.11	0.17
NV	0.96	0.04	0.07	0.98	0.11	0.07
NY	0.95	0.00	0.08	1.00	0.08	0.00
OH	0.87	0.01	0.15	0.99	0.15	0.01
OK	1.00	0.02	0.00	0.99	0.02	0.04
OR	0.97	0.01	0.08	1.00	0.09	0.02
PA	0.96	0.00	0.05	1.00	0.05	0.00
RI	0.86	0.00	0.28	1.00	0.28	0.00
SC	1.00	0.01	0.01	1.00	0.01	0.01
SD	0.99	0.00	0.18	1.00	0.18	0.00
TN	1.00	0.01	0.00	0.99	0.02	0.03
TX	1.00	0.04	0.02	0.99	0.05	0.07
UT	0.82	0.13	0.17	0.86	0.26	0.23
VA	0.83	0.00	0.42	1.00	0.42	0.00
VT	0.94	0.01	0.09	0.99	0.11	0.03
WA	0.90	0.02	0.04	0.95	0.05	0.04
WI	1.00	0.01	0.00	1.00	0.01	0.01
WV	0.97	0.00	0.26	1.00	0.26	0.00
WY	0.91	0.04	0.51	1.00	0.52	0.08
US	0.99	0.02	0.06	1.00	0.08	0.04

Table S5 Continued

State	P-P300	P-U300	P-NP300	P-NU300	P-AllGW300	-F1score30
AL	0.02	0.01	1.00	1.00	0.99	0.01
AR	0.74	0.13	0.81	0.99	0.80	0.22
AZ	0.24	0.05	0.98	1.00	0.97	0.09
CA	0.31	0.03	0.92	0.99	0.91	0.05
CO	0.31	0.05	0.95	0.99	0.95	0.09
CT	0.01	0.01	1.00	1.00	1.00	0.01
DC	0.00	0.00	1.00	1.00	1.00	0.00
DE	0.39	0.20	0.82	0.92	0.78	0.26
FL	0.15	0.20	0.96	0.94	0.90	0.17
GA	0.59	0.05	0.91	1.00	0.90	0.09
IA	0.21	0.02	0.99	1.00	0.99	0.03
ID	0.17	0.04	0.96	0.99	0.95	0.06
IL	0.20	0.02	0.97	1.00	0.96	0.03
IN	0.26	0.02	0.96	1.00	0.95	0.03
KS	0.39	0.03	0.90	0.99	0.89	0.06
KY	0.02	0.01	1.00	1.00	0.99	0.01
LA	0.35	0.05	0.92	0.99	0.92	0.09
MA	0.00	0.00	1.00	0.97	0.97	0.00
MD	0.12	0.08	0.97	0.98	0.95	0.09
ME	0.00	0.00	1.00	1.00	1.00	0.00
MI	0.11	0.02	0.97	0.99	0.96	0.04
MN	0.32	0.03	0.97	1.00	0.96	0.05
MO	0.78	0.03	0.95	1.00	0.95	0.05
MS	0.69	0.12	0.91	0.99	0.91	0.20
MT	0.01	0.01	1.00	1.00	1.00	0.01
NC	0.06	0.00	1.00	1.00	1.00	0.01

ND	0.11	0.01	0.99	1.00	0.99	0.02
NE	0.77	0.08	0.73	0.99	0.73	0.15
NH	0.00	0.00	1.00	1.00	1.00	0.00
NJ	0.14	0.15	0.95	0.95	0.91	0.14
NM	0.40	0.11	0.97	0.99	0.96	0.17
NV	0.34	0.06	0.99	1.00	0.99	0.10
NY	0.05	0.08	1.00	1.00	1.00	0.06
OH	0.00	0.00	1.00	0.99	0.99	0.00
OK	0.11	0.03	0.97	0.99	0.96	0.05
OR	0.18	0.02	0.98	1.00	0.98	0.03
PA	0.00	0.00	1.00	1.00	1.00	0.00
RI	0.00	0.00	1.00	1.00	1.00	0.00
SC	0.11	0.01	0.98	1.00	0.98	0.02
SD	0.14	0.01	0.98	1.00	0.98	0.02
TN	0.02	0.03	0.99	0.99	0.98	0.02
TX	0.34	0.05	0.93	0.99	0.92	0.09
UT	0.07	0.13	0.99	0.98	0.98	0.09
VA	0.06	0.04	1.00	1.00	1.00	0.04
VT	0.00	0.00	1.00	1.00	1.00	0.00
WA	0.07	0.04	0.97	0.98	0.95	0.05
WI	0.42	0.03	0.97	1.00	0.97	0.06
WV	0.00	0.00	1.00	1.00	1.00	0.00
WY	0.19	0.04	0.99	1.00	0.99	0.07
US	0.34	0.06	0.96	0.99	0.95	0.10

Table S5 Continued

State	G-P500	G-U500	G-NP500	G-NU500	G-AllGW500	-F1score50
AL	0.98	0.02	0.09	1.00	0.11	0.03
AR	1.00	0.32	0.01	1.00	0.33	0.49
AZ	1.00	0.12	0.01	0.93	0.13	0.22
CA	1.00	0.08	0.00	0.94	0.08	0.14
CO	1.00	0.08	0.00	0.97	0.09	0.16
CT	0.94	0.01	0.12	0.99	0.13	0.02
DC	0.00	0.00	0.42	1.00	0.42	0.00
DE	1.00	0.41	0.00	0.96	0.41	0.58
FL	1.00	0.37	0.00	0.64	0.37	0.54
GA	1.00	0.12	0.00	1.00	0.12	0.21
IA	1.00	0.00	0.01	1.00	0.01	0.01
ID	0.99	0.10	0.01	0.88	0.11	0.19
IL	1.00	0.01	0.00	0.97	0.01	0.02
IN	1.00	0.01	0.00	1.00	0.01	0.02
KS	1.00	0.05	0.00	1.00	0.05	0.09
KY	1.00	0.02	0.00	1.00	0.02	0.03
LA	1.00	0.12	0.00	0.97	0.12	0.21
MA	0.68	0.06	0.24	0.91	0.27	0.11
MD	1.00	0.13	0.01	0.96	0.14	0.23
ME	1.00	0.01	0.02	1.00	0.02	0.01
MI	1.00	0.03	0.00	0.97	0.04	0.07
MN	1.00	0.01	0.00	0.97	0.01	0.02
MO	1.00	0.02	0.02	1.00	0.03	0.03
MS	1.00	0.25	0.00	1.00	0.25	0.40
MT	0.70	0.00	0.22	0.99	0.22	0.01
NC	0.99	0.00	0.13	1.00	0.13	0.01

ND	1.00	0.00	0.06	1.00	0.06	0.01
NE	1.00	0.19	0.00	0.96	0.19	0.31
NH	0.96	0.01	0.10	1.00	0.10	0.01
NJ	1.00	0.22	0.00	0.83	0.23	0.37
NM	1.00	0.24	0.02	0.97	0.25	0.39
NV	0.98	0.11	0.03	0.92	0.13	0.20
NY	0.97	0.01	0.02	0.99	0.03	0.01
OH	0.92	0.02	0.11	0.98	0.13	0.04
OK	1.00	0.06	0.00	0.98	0.06	0.11
OR	0.99	0.03	0.04	0.99	0.07	0.06
PA	0.99	0.01	0.01	1.00	0.02	0.01
RI	0.35	0.00	0.15	1.00	0.15	0.00
SC	1.00	0.02	0.00	0.99	0.02	0.04
SD	0.99	0.01	0.16	1.00	0.16	0.01
TN	1.00	0.04	0.00	0.98	0.04	0.08
TX	1.00	0.10	0.01	0.97	0.10	0.18
UT	0.96	0.32	0.05	0.74	0.34	0.48
VA	0.92	0.00	0.29	1.00	0.29	0.01
VT	0.99	0.04	0.03	0.99	0.07	0.08
WA	0.97	0.06	0.01	0.84	0.07	0.11
WI	1.00	0.02	0.00	0.99	0.02	0.04
WV	0.86	0.00	0.13	1.00	0.13	0.00
WY	0.95	0.09	0.45	0.99	0.48	0.17
US	0.99	0.05	0.04	0.99	0.08	0.10

Table S5 Continued

State	P-P500	P-U500	P-NP500	P-NU500	P-AllGW500	-F1score50
AL	0.04	0.03	0.99	0.99	0.98	0.03
AR	0.85	0.33	0.81	0.98	0.81	0.48
AZ	0.36	0.16	0.97	0.99	0.96	0.22
CA	0.41	0.09	0.89	0.98	0.88	0.14
CO	0.39	0.13	0.93	0.98	0.92	0.20
CT	0.03	0.06	1.00	1.00	1.00	0.04
DC	0.00	0.00	1.00	1.00	1.00	0.00
DE	0.58	0.44	0.77	0.85	0.72	0.50
FL	0.22	0.42	0.94	0.86	0.82	0.29
GA	0.75	0.12	0.86	0.99	0.86	0.21
IA	0.29	0.04	0.98	1.00	0.98	0.07
ID	0.25	0.12	0.95	0.98	0.93	0.16
IL	0.26	0.05	0.95	0.99	0.94	0.08
IN	0.38	0.05	0.93	0.99	0.92	0.08
KS	0.50	0.09	0.86	0.98	0.85	0.15
KY	0.04	0.03	0.99	0.99	0.98	0.03
LA	0.48	0.15	0.90	0.98	0.88	0.23
MA	0.00	0.00	1.00	0.92	0.92	0.00
MD	0.19	0.19	0.95	0.95	0.91	0.19
ME	0.00	0.00	1.00	1.00	1.00	0.00
MI	0.19	0.07	0.95	0.98	0.93	0.10
MN	0.44	0.06	0.94	0.99	0.94	0.11
MO	0.86	0.06	0.93	1.00	0.93	0.12
MS	0.80	0.30	0.91	0.99	0.90	0.43
MT	0.01	0.03	1.00	1.00	1.00	0.02
NC	0.08	0.01	0.99	1.00	0.99	0.01

ND	0.15	0.02	0.98	1.00	0.98	0.04
NE	0.86	0.21	0.67	0.98	0.69	0.34
NH	0.00	0.00	1.00	1.00	1.00	0.00
NJ	0.21	0.31	0.93	0.88	0.83	0.25
NM	0.50	0.25	0.96	0.99	0.95	0.33
NV	0.41	0.14	0.99	1.00	0.99	0.21
NY	0.07	0.15	1.00	0.99	0.99	0.10
OH	0.00	0.01	1.00	0.98	0.98	0.00
OK	0.17	0.09	0.95	0.98	0.93	0.12
OR	0.29	0.05	0.98	1.00	0.97	0.08
PA	0.00	0.01	1.00	1.00	1.00	0.00
RI	0.00	0.00	1.00	1.00	1.00	0.00
SC	0.17	0.03	0.96	0.99	0.95	0.05
SD	0.21	0.02	0.97	1.00	0.97	0.04
TN	0.04	0.07	0.99	0.98	0.96	0.05
TX	0.46	0.13	0.90	0.98	0.89	0.20
UT	0.11	0.31	0.99	0.96	0.95	0.17
VA	0.10	0.08	1.00	1.00	1.00	0.09
VT	0.00	0.00	1.00	0.99	0.99	0.00
WA	0.13	0.12	0.95	0.96	0.91	0.12
WI	0.53	0.08	0.95	1.00	0.94	0.13
WV	0.00	0.00	1.00	1.00	1.00	0.00
WY	0.26	0.10	0.99	1.00	0.99	0.14
US	0.43	0.15	0.94	0.99	0.93	0.22

Table S5 Continued

State	G-P1000	G-U1000	G-NP1000	G-NU1000	G-AllGW1000	F1score100
AL	0.98	0.05	0.06	0.99	0.11	0.10
AR	1.00	0.68	0.01	0.99	0.68	0.81
AZ	1.00	0.35	0.01	0.88	0.35	0.51
CA	1.00	0.26	0.00	0.89	0.26	0.41
CO	1.00	0.20	0.00	0.97	0.20	0.33
CT	0.99	0.04	0.05	0.99	0.09	0.07
DC	0.00	0.00	0.40	1.00	0.40	0.00
DE	1.00	0.76	0.00	0.98	0.76	0.87
FL	1.00	0.69	0.00	0.37	0.69	0.82
GA	1.00	0.31	0.00	0.99	0.31	0.47
IA	1.00	0.01	0.01	1.00	0.02	0.02
ID	1.00	0.29	0.00	0.76	0.30	0.45
IL	1.00	0.03	0.00	0.85	0.03	0.06
IN	1.00	0.04	0.00	1.00	0.04	0.07
KS	1.00	0.14	0.00	1.00	0.14	0.24
KY	1.00	0.05	0.00	0.98	0.05	0.10
LA	1.00	0.32	0.00	0.94	0.33	0.49
MA	0.82	0.17	0.11	0.75	0.24	0.28
MD	1.00	0.35	0.01	0.95	0.35	0.52
ME	1.00	0.01	0.01	1.00	0.03	0.03
MI	1.00	0.11	0.00	0.94	0.11	0.20
MN	1.00	0.03	0.00	0.91	0.03	0.06
MO	1.00	0.04	0.01	1.00	0.05	0.08
MS	1.00	0.53	0.00	0.98	0.53	0.69
MT	0.92	0.01	0.05	0.98	0.06	0.03
NC	0.99	0.01	0.07	1.00	0.08	0.02

ND	1.00	0.01	0.06	1.00	0.06	0.02
NE	1.00	0.45	0.00	0.92	0.45	0.62
NH	1.00	0.02	0.05	1.00	0.06	0.04
NJ	1.00	0.48	0.00	0.64	0.48	0.65
NM	1.00	0.46	0.03	0.95	0.47	0.63
NV	0.98	0.28	0.02	0.76	0.29	0.43
NY	0.99	0.02	0.00	0.95	0.02	0.04
OH	0.93	0.08	0.10	0.95	0.16	0.14
OK	1.00	0.16	0.00	0.96	0.16	0.27
OR	0.99	0.09	0.03	0.98	0.12	0.17
PA	1.00	0.02	0.00	0.99	0.02	0.04
RI	0.68	0.00	0.08	0.99	0.08	0.00
SC	1.00	0.06	0.00	0.98	0.06	0.12
SD	1.00	0.02	0.15	1.00	0.16	0.04
TN	1.00	0.14	0.00	0.96	0.14	0.24
TX	1.00	0.25	0.00	0.89	0.25	0.39
UT	0.99	0.60	0.02	0.59	0.60	0.75
VA	0.96	0.01	0.20	1.00	0.21	0.02
VT	1.00	0.11	0.01	0.97	0.12	0.21
WA	0.99	0.15	0.00	0.74	0.16	0.27
WI	1.00	0.06	0.00	0.95	0.06	0.11
WV	0.97	0.00	0.05	1.00	0.05	0.00
WY	0.96	0.18	0.39	0.99	0.46	0.30
US	1.00	0.13	0.03	0.98	0.15	0.23

Table S5 Continued

State	P-P1000	P-U1000	P-NP1000	P-NU1000	P-AllGW1000	F1score100
AL	0.08	0.09	0.98	0.98	0.96	0.08
AR	0.92	0.65	0.86	0.98	0.87	0.76
AZ	0.49	0.40	0.97	0.98	0.95	0.44
CA	0.55	0.28	0.87	0.96	0.85	0.37
CO	0.48	0.27	0.91	0.96	0.88	0.34
CT	0.07	0.18	1.00	0.99	0.99	0.10
DC	0.00	0.00	1.00	1.00	1.00	0.00
DE	0.76	0.77	0.78	0.77	0.77	0.77
FL	0.32	0.70	0.93	0.72	0.72	0.44
GA	0.86	0.27	0.80	0.98	0.80	0.41
IA	0.41	0.09	0.96	0.99	0.96	0.15
ID	0.36	0.31	0.93	0.95	0.89	0.33
IL	0.37	0.10	0.90	0.98	0.89	0.16
IN	0.52	0.11	0.87	0.98	0.86	0.18
KS	0.65	0.22	0.79	0.96	0.78	0.32
KY	0.08	0.09	0.98	0.98	0.96	0.09
LA	0.61	0.37	0.87	0.95	0.84	0.46
MA	0.00	0.00	1.00	0.82	0.82	0.00
MD	0.28	0.43	0.92	0.86	0.82	0.34
ME	0.00	0.00	1.00	1.00	0.99	0.00
MI	0.30	0.17	0.91	0.95	0.87	0.22
MN	0.57	0.13	0.89	0.99	0.88	0.21
MO	0.92	0.13	0.91	1.00	0.91	0.22
MS	0.84	0.55	0.91	0.98	0.90	0.67
MT	0.02	0.09	1.00	0.99	0.99	0.04
NC	0.16	0.02	0.98	1.00	0.98	0.04

ND	0.22	0.04	0.96	0.99	0.95	0.07
NE	0.92	0.44	0.62	0.96	0.69	0.60
NH	0.00	0.00	1.00	1.00	1.00	0.00
NJ	0.32	0.55	0.88	0.74	0.71	0.40
NM	0.59	0.43	0.95	0.97	0.93	0.50
NV	0.48	0.31	0.99	0.99	0.98	0.38
NY	0.08	0.22	1.00	0.99	0.98	0.12
OH	0.00	0.05	1.00	0.93	0.93	0.00
OK	0.26	0.21	0.91	0.93	0.86	0.23
OR	0.42	0.13	0.96	0.99	0.95	0.19
PA	0.01	0.03	1.00	0.99	0.99	0.01
RI	0.00	0.00	1.00	1.00	1.00	0.00
SC	0.28	0.07	0.91	0.98	0.89	0.12
SD	0.32	0.05	0.95	0.99	0.94	0.09
TN	0.07	0.20	0.97	0.92	0.90	0.10
TX	0.57	0.28	0.87	0.96	0.84	0.38
UT	0.17	0.56	0.99	0.92	0.91	0.26
VA	0.17	0.17	1.00	1.00	1.00	0.17
VT	0.00	0.00	1.00	0.96	0.96	0.00
WA	0.21	0.26	0.92	0.90	0.84	0.23
WI	0.62	0.14	0.90	0.99	0.89	0.23
WV	0.00	0.00	1.00	1.00	1.00	0.00
WY	0.35	0.20	0.99	0.99	0.98	0.26
US	0.52	0.30	0.92	0.97	0.90	0.38

Table S5 Continued

State	G-P2000	G-U2000	G-NP2000	G-NU2000	G-AllGW2000	F1score200
AL	0.99	0.15	0.05	0.96	0.18	0.25
AR	1.00	0.84	0.01	0.99	0.84	0.91
AZ	1.00	0.61	0.01	0.80	0.61	0.76
CA	1.00	0.66	0.00	0.77	0.66	0.79
CO	1.00	0.35	0.00	0.97	0.35	0.52
CT	1.00	0.09	0.02	1.00	0.11	0.17
DC	0.00	0.00	0.38	1.00	0.38	0.00
DE	1.00	0.94	0.00	1.00	0.94	0.97
FL	1.00	0.95	0.00	0.22	0.95	0.97
GA	1.00	0.55	0.00	0.99	0.55	0.71
IA	1.00	0.02	0.01	1.00	0.03	0.05
ID	1.00	0.56	0.00	0.55	0.56	0.72
IL	1.00	0.07	0.00	0.60	0.07	0.14
IN	1.00	0.10	0.00	1.00	0.10	0.18
KS	1.00	0.27	0.00	1.00	0.27	0.42
KY	1.00	0.15	0.00	0.93	0.15	0.26
LA	1.00	0.61	0.00	0.86	0.61	0.76
MA	0.92	0.35	0.04	0.51	0.36	0.50
MD	1.00	0.70	0.01	0.93	0.70	0.82
ME	1.00	0.03	0.01	1.00	0.04	0.07
MI	1.00	0.30	0.00	0.91	0.30	0.46
MN	1.00	0.07	0.00	0.90	0.07	0.13
MO	1.00	0.08	0.01	1.00	0.09	0.16
MS	1.00	0.67	0.00	0.95	0.67	0.81
MT	0.98	0.04	0.01	0.95	0.05	0.08
NC	1.00	0.03	0.04	1.00	0.07	0.05

ND	1.00	0.02	0.05	1.00	0.07	0.05
NE	1.00	0.64	0.01	0.88	0.64	0.78
NH	1.00	0.05	0.02	1.00	0.07	0.10
NJ	1.00	0.78	0.00	0.47	0.78	0.88
NM	1.00	0.61	0.03	0.94	0.61	0.76
NV	0.98	0.47	0.02	0.52	0.47	0.63
NY	0.99	0.05	0.00	0.75	0.05	0.09
OH	0.94	0.25	0.09	0.83	0.30	0.39
OK	1.00	0.36	0.00	0.87	0.36	0.53
OR	1.00	0.22	0.03	0.95	0.24	0.36
PA	1.00	0.07	0.00	0.96	0.07	0.12
RI	0.96	0.03	0.04	0.98	0.07	0.05
SC	1.00	0.17	0.00	0.93	0.17	0.29
SD	1.00	0.06	0.14	1.00	0.19	0.11
TN	1.00	0.38	0.00	0.90	0.38	0.55
TX	1.00	0.46	0.00	0.77	0.46	0.63
UT	0.99	0.82	0.01	0.30	0.82	0.90
VA	0.97	0.03	0.15	1.00	0.17	0.06
VT	1.00	0.30	0.00	0.96	0.30	0.46
WA	1.00	0.33	0.00	0.53	0.33	0.49
WI	1.00	0.12	0.00	0.87	0.12	0.21
WV	0.99	0.01	0.02	1.00	0.02	0.01
WY	0.95	0.29	0.36	0.97	0.49	0.44
US	1.00	0.23	0.02	0.96	0.25	0.38

Table S5 Continued

State	P-P2000	P-U2000	P-NP2000	P-NU2000	P-AllGW2000	-F1score200
AL	0.16	0.26	0.97	0.94	0.91	0.20
AR	0.95	0.78	0.87	0.97	0.90	0.85
AZ	0.60	0.67	0.97	0.96	0.93	0.63
CA	0.68	0.65	0.90	0.91	0.85	0.67
CO	0.58	0.40	0.86	0.92	0.82	0.47
CT	0.14	0.49	1.00	0.98	0.97	0.22
DC	0.00	0.00	1.00	1.00	1.00	0.00
DE	0.86	0.94	0.88	0.75	0.87	0.90
FL	0.44	0.90	0.94	0.57	0.66	0.59
GA	0.93	0.48	0.75	0.98	0.78	0.63
IA	0.53	0.13	0.92	0.99	0.91	0.21
ID	0.47	0.56	0.93	0.90	0.85	0.51
IL	0.47	0.17	0.81	0.95	0.78	0.25
IN	0.66	0.21	0.78	0.96	0.77	0.32
KS	0.78	0.34	0.67	0.93	0.69	0.47
KY	0.16	0.27	0.96	0.93	0.90	0.20
LA	0.68	0.63	0.87	0.89	0.82	0.66
MA	0.00	0.07	1.00	0.66	0.66	0.00
MD	0.37	0.71	0.90	0.69	0.69	0.48
ME	0.00	0.00	0.99	0.99	0.98	0.00
MI	0.46	0.40	0.86	0.89	0.79	0.42
MN	0.67	0.20	0.80	0.97	0.79	0.31
MO	0.92	0.18	0.86	1.00	0.86	0.30
MS	0.79	0.63	0.89	0.95	0.88	0.70
MT	0.04	0.20	1.00	0.98	0.97	0.07
NC	0.25	0.05	0.96	0.99	0.95	0.08

ND	0.34	0.07	0.91	0.98	0.89	0.12
NE	0.95	0.60	0.54	0.94	0.71	0.73
NH	0.00	0.00	1.00	0.99	0.99	0.00
NJ	0.44	0.78	0.84	0.53	0.61	0.56
NM	0.63	0.53	0.94	0.96	0.90	0.58
NV	0.49	0.47	0.98	0.98	0.96	0.48
NY	0.08	0.28	0.99	0.96	0.95	0.12
OH	0.01	0.26	0.99	0.79	0.79	0.02
OK	0.37	0.42	0.85	0.83	0.74	0.39
OR	0.56	0.26	0.94	0.98	0.93	0.35
PA	0.02	0.22	1.00	0.97	0.96	0.04
RI	0.00	0.00	0.99	0.99	0.99	0.00
SC	0.40	0.18	0.83	0.94	0.80	0.25
SD	0.49	0.11	0.89	0.98	0.88	0.18
TN	0.12	0.46	0.95	0.76	0.74	0.19
TX	0.62	0.45	0.82	0.90	0.78	0.52
UT	0.22	0.77	0.99	0.86	0.85	0.35
VA	0.26	0.36	1.00	0.99	0.99	0.30
VT	0.00	0.00	1.00	0.88	0.88	0.00
WA	0.29	0.43	0.88	0.80	0.74	0.35
WI	0.67	0.22	0.82	0.97	0.81	0.33
WV	0.00	0.00	1.00	1.00	1.00	0.00
WY	0.42	0.32	0.98	0.99	0.97	0.36
US	0.56	0.44	0.89	0.93	0.85	0.49

Table S5 Continued

State	G-P3000	G-U3000	G-NP3000	G-NU3000	G-AllGW3000	F1score300
AL	0.99	0.23	0.04	0.93	0.25	0.37
AR	1.00	0.87	0.00	0.98	0.87	0.93
AZ	1.00	0.72	0.01	0.79	0.72	0.84
CA	1.00	0.75	0.01	0.74	0.75	0.86
CO	1.00	0.45	0.00	0.96	0.45	0.62
CT	1.00	0.14	0.01	0.98	0.15	0.25
DC	0.00	0.00	0.32	0.99	0.32	0.00
DE	1.00	0.98	0.00	1.00	0.98	0.99
FL	1.00	0.98	0.00	0.08	0.98	0.99
GA	1.00	0.67	0.00	0.99	0.67	0.80
IA	1.00	0.04	0.01	1.00	0.04	0.07
ID	1.00	0.69	0.00	0.45	0.69	0.82
IL	1.00	0.11	0.00	0.37	0.11	0.21
IN	1.00	0.16	0.00	0.00	0.16	0.27
KS	1.00	0.34	0.00	1.00	0.34	0.51
KY	1.00	0.24	0.00	0.94	0.24	0.39
LA	1.00	0.75	0.00	0.79	0.75	0.86
MA	0.97	0.50	0.03	0.45	0.50	0.66
MD	1.00	0.86	0.01	0.91	0.86	0.92
ME	1.00	0.06	0.01	0.99	0.07	0.12
MI	1.00	0.46	0.00	0.83	0.46	0.63
MN	1.00	0.11	0.00	0.90	0.11	0.19
MO	1.00	0.12	0.01	1.00	0.13	0.21
MS	1.00	0.71	0.00	0.92	0.71	0.83
MT	0.99	0.07	0.01	0.93	0.08	0.13
NC	0.99	0.04	0.04	0.99	0.07	0.08

ND	1.00	0.04	0.05	1.00	0.09	0.07
NE	1.00	0.73	0.01	0.86	0.73	0.84
NH	1.00	0.09	0.02	1.00	0.10	0.16
NJ	1.00	0.90	0.00	0.33	0.90	0.95
NM	1.00	0.65	0.04	0.90	0.65	0.79
NV	0.98	0.55	0.02	0.55	0.55	0.71
NY	0.99	0.09	0.00	0.62	0.09	0.16
OH	0.94	0.43	0.09	0.71	0.45	0.59
OK	1.00	0.51	0.00	0.87	0.51	0.68
OR	1.00	0.33	0.03	0.93	0.34	0.49
PA	1.00	0.12	0.00	0.92	0.12	0.22
RI	0.99	0.05	0.03	0.98	0.08	0.10
SC	1.00	0.29	0.00	0.81	0.29	0.45
SD	0.99	0.09	0.14	1.00	0.21	0.17
TN	1.00	0.58	0.00	0.80	0.58	0.73
TX	1.00	0.57	0.00	0.72	0.57	0.73
UT	0.99	0.90	0.02	0.26	0.90	0.95
VA	0.97	0.04	0.13	0.99	0.16	0.08
VT	1.00	0.46	0.00	0.88	0.46	0.63
WA	1.00	0.47	0.00	0.40	0.47	0.64
WI	1.00	0.17	0.00	0.80	0.17	0.30
WV	1.00	0.01	0.01	1.00	0.02	0.03
WY	0.94	0.37	0.36	0.94	0.52	0.53
US	1.00	0.30	0.02	0.94	0.31	0.46

Table S5 Continued

State	P-P3000	P-U3000	P-NP3000	P-NU3000	P-AllGW3000	F1score300
AL	0.19	0.40	0.96	0.90	0.87	0.26
AR	0.95	0.79	0.86	0.97	0.89	0.86
AZ	0.64	0.76	0.97	0.95	0.92	0.69
CA	0.69	0.74	0.90	0.88	0.84	0.72
CO	0.63	0.48	0.82	0.90	0.78	0.54
CT	0.16	0.78	1.00	0.95	0.95	0.26
DC	0.00	0.00	1.00	1.00	1.00	0.00
DE	0.88	0.96	0.90	0.72	0.89	0.92
FL	0.50	0.95	0.95	0.50	0.66	0.66
GA	0.95	0.59	0.73	0.97	0.80	0.73
IA	0.56	0.16	0.89	0.98	0.87	0.24
ID	0.51	0.67	0.92	0.85	0.82	0.58
IL	0.53	0.21	0.73	0.92	0.70	0.30
IN	0.72	0.28	0.71	0.94	0.71	0.40
KS	0.85	0.39	0.57	0.92	0.64	0.54
KY	0.20	0.40	0.95	0.87	0.84	0.27
LA	0.70	0.75	0.87	0.85	0.81	0.72
MA	0.00	0.62	1.00	0.56	0.56	0.01
MD	0.42	0.85	0.91	0.57	0.65	0.56
ME	0.00	0.00	0.98	0.98	0.96	0.00
MI	0.54	0.56	0.84	0.83	0.76	0.55
MN	0.71	0.25	0.74	0.95	0.74	0.37
MO	0.91	0.19	0.81	0.99	0.82	0.32
MS	0.72	0.63	0.87	0.91	0.83	0.67
MT	0.05	0.29	0.99	0.96	0.95	0.09
NC	0.30	0.08	0.94	0.99	0.93	0.12

ND	0.41	0.10	0.86	0.98	0.84	0.15
NE	0.96	0.68	0.50	0.92	0.74	0.79
NH	0.00	0.00	1.00	0.99	0.99	0.00
NJ	0.52	0.91	0.86	0.40	0.61	0.66
NM	0.63	0.58	0.92	0.94	0.88	0.60
NV	0.50	0.54	0.97	0.97	0.95	0.52
NY	0.08	0.36	0.99	0.93	0.92	0.13
OH	0.02	0.49	0.99	0.65	0.64	0.04
OK	0.45	0.56	0.81	0.73	0.68	0.50
OR	0.62	0.34	0.92	0.97	0.91	0.44
PA	0.03	0.39	1.00	0.93	0.93	0.06
RI	0.00	0.00	0.99	0.98	0.97	0.00
SC	0.47	0.29	0.79	0.89	0.74	0.36
SD	0.59	0.16	0.85	0.98	0.84	0.25
TN	0.16	0.66	0.94	0.61	0.61	0.25
TX	0.63	0.53	0.78	0.85	0.74	0.58
UT	0.25	0.86	0.99	0.80	0.81	0.39
VA	0.31	0.49	0.99	0.99	0.98	0.38
VT	0.00	0.00	1.00	0.77	0.77	0.00
WA	0.35	0.55	0.85	0.72	0.69	0.43
WI	0.70	0.29	0.77	0.95	0.76	0.41
WV	0.00	0.00	1.00	1.00	0.99	0.00
WY	0.44	0.40	0.97	0.98	0.95	0.42
US	0.57	0.50	0.87	0.90	0.81	0.53

Table S5 Continued

State	G-P5000	G-U5000	G-NP5000	G-NU5000	G-AllGW5000	F1score500
AL	0.99	0.35	0.04	0.87	0.37	0.52
AR	1.00	0.89	0.00	0.96	0.89	0.94
AZ	1.00	0.82	0.02	0.79	0.81	0.90
CA	1.00	0.85	0.01	0.63	0.85	0.92
CO	1.00	0.59	0.00	0.95	0.59	0.74
CT	1.00	0.25	0.00	0.76	0.25	0.40
DC	1.00	0.03	0.23	1.00	0.25	0.06
DE	1.00	0.99	0.03	1.00	0.99	0.99
FL	1.00	1.00	0.01	0.05	1.00	1.00
GA	1.00	0.77	0.00	1.00	0.77	0.87
IA	1.00	0.07	0.01	1.00	0.07	0.12
ID	1.00	0.83	0.01	0.34	0.83	0.91
IL	1.00	0.20	0.00	0.48	0.20	0.34
IN	1.00	0.26	0.00	1.00	0.26	0.42
KS	1.00	0.44	0.00	1.00	0.44	0.61
KY	1.00	0.42	0.00	0.65	0.42	0.59
LA	1.00	0.88	0.00	0.69	0.88	0.94
MA	0.99	0.70	0.02	0.40	0.69	0.82
MD	1.00	0.94	0.02	0.76	0.94	0.97
ME	1.00	0.11	0.01	1.00	0.11	0.19
MI	1.00	0.72	0.00	0.77	0.72	0.83
MN	1.00	0.18	0.00	0.58	0.18	0.31
MO	1.00	0.16	0.01	0.99	0.17	0.28
MS	1.00	0.75	0.00	0.79	0.75	0.86
MT	1.00	0.14	0.00	0.89	0.14	0.24
NC	0.98	0.08	0.03	0.95	0.10	0.14

ND	0.99	0.07	0.05	0.99	0.12	0.13
NE	1.00	0.82	0.01	0.82	0.82	0.90
NH	1.00	0.17	0.01	1.00	0.18	0.29
NJ	1.00	0.98	0.01	0.43	0.98	0.99
NM	1.00	0.70	0.04	0.88	0.71	0.83
NV	0.98	0.66	0.03	0.46	0.66	0.79
NY	1.00	0.19	0.00	0.52	0.19	0.31
OH	0.95	0.72	0.12	0.51	0.71	0.82
OK	1.00	0.74	0.00	0.82	0.74	0.85
OR	1.00	0.50	0.03	0.91	0.51	0.67
PA	1.00	0.25	0.00	0.84	0.25	0.40
RI	1.00	0.11	0.01	1.00	0.12	0.20
SC	1.00	0.49	0.00	0.79	0.49	0.66
SD	0.99	0.17	0.15	0.99	0.27	0.29
TN	1.00	0.84	0.00	0.60	0.84	0.91
TX	1.00	0.69	0.00	0.63	0.69	0.82
UT	0.99	0.96	0.03	0.23	0.95	0.98
VA	0.96	0.08	0.10	0.97	0.16	0.14
VT	1.00	0.70	0.00	0.94	0.70	0.82
WA	1.00	0.68	0.00	0.29	0.68	0.81
WI	1.00	0.30	0.00	0.90	0.30	0.46
WV	1.00	0.04	0.00	1.00	0.04	0.07
WY	0.91	0.49	0.36	0.86	0.58	0.64
US	1.00	0.40	0.02	0.90	0.41	0.57

Table S5 Continued

State	P-P5000	P-U5000	P-NP5000	P-NU5000	P-AllGW5000	F1score500
AL	0.23	0.59	0.95	0.81	0.79	0.33
AR	0.93	0.81	0.85	0.94	0.88	0.86
AZ	0.65	0.84	0.97	0.91	0.90	0.73
CA	0.67	0.83	0.91	0.81	0.82	0.74
CO	0.71	0.59	0.77	0.85	0.75	0.64
CT	0.16	0.93	1.00	0.91	0.91	0.28
DC	0.00	0.00	1.00	0.98	0.98	0.00
DE	0.90	0.98	0.91	0.68	0.90	0.94
FL	0.58	0.99	0.98	0.41	0.67	0.73
GA	0.95	0.73	0.75	0.96	0.84	0.83
IA	0.62	0.20	0.83	0.97	0.81	0.30
ID	0.54	0.81	0.92	0.77	0.78	0.65
IL	0.64	0.31	0.60	0.86	0.61	0.42
IN	0.75	0.39	0.64	0.89	0.66	0.51
KS	0.93	0.47	0.45	0.92	0.61	0.62
KY	0.24	0.61	0.94	0.76	0.75	0.34
LA	0.69	0.86	0.88	0.74	0.79	0.76
MA	0.01	1.00	1.00	0.39	0.39	0.02
MD	0.45	0.94	0.93	0.40	0.58	0.61
ME	0.00	0.00	0.97	0.96	0.93	0.00
MI	0.65	0.79	0.87	0.77	0.77	0.71
MN	0.75	0.35	0.66	0.92	0.68	0.48
MO	0.90	0.21	0.74	0.99	0.75	0.34
MS	0.62	0.65	0.82	0.80	0.75	0.63
MT	0.07	0.40	0.99	0.91	0.90	0.11
NC	0.39	0.14	0.91	0.98	0.90	0.20

ND	0.51	0.15	0.78	0.96	0.76	0.23
NE	0.96	0.77	0.45	0.84	0.79	0.86
NH	0.00	0.00	1.00	0.97	0.97	0.00
NJ	0.61	0.97	0.89	0.30	0.66	0.75
NM	0.62	0.63	0.90	0.90	0.84	0.62
NV	0.49	0.61	0.96	0.94	0.91	0.54
NY	0.07	0.47	0.99	0.86	0.85	0.13
OH	0.03	0.86	0.99	0.42	0.42	0.06
OK	0.56	0.76	0.77	0.57	0.65	0.65
OR	0.72	0.47	0.91	0.97	0.89	0.57
PA	0.04	0.72	1.00	0.86	0.86	0.08
RI	0.00	0.00	0.98	0.96	0.94	0.00
SC	0.55	0.51	0.76	0.79	0.70	0.53
SD	0.70	0.23	0.78	0.97	0.78	0.35
TN	0.20	0.92	0.96	0.38	0.46	0.33
TX	0.64	0.64	0.73	0.73	0.69	0.64
UT	0.28	0.94	0.99	0.69	0.72	0.43
VA	0.27	0.66	1.00	0.97	0.97	0.38
VT	0.00	0.00	1.00	0.56	0.56	0.00
WA	0.45	0.71	0.83	0.62	0.65	0.55
WI	0.75	0.42	0.72	0.91	0.73	0.54
WV	0.02	0.08	1.00	0.99	0.99	0.04
WY	0.45	0.53	0.97	0.96	0.93	0.49
US	0.58	0.60	0.84	0.84	0.77	0.59

Table S6 Groundwater irrigation prediction accuracy against the Indian farmers survey by State

State	G-P300	G-U300	G-NP300	G-NU300	G-AllGW300	[illegible]-F1score30[illegible]
Bihar	1.00	0.91	0.01	0.31	0.91	0.95
Chhattisgarh	0.99	0.26	0.06	0.94	0.29	0.41
Jharkhand	0.00	0.00	0.00	0.00	0.00	0.00
Uttar Pradesh	1.00	0.90	0.01	1.00	0.90	0.95
Andhra Pradesh	0.99	0.24	0.03	0.89	0.26	0.39
Haryana	1.00	0.99	0.00	0.00	0.99	1.00
Orissa	0.97	0.24	0.16	0.95	0.33	0.38
Punjab	1.00	1.00	0.00	0.00	1.00	1.00
West Bengal	1.00	0.66	0.12	0.97	0.68	0.80
India	1.00	0.71	0.07	0.93	0.71	0.83

Table S6 Continued

State	P-P300	P-U300	P-NP300	P-NU300	P-AllGW300	[illegible]-F1score30[illegible]
Bihar	0.55	0.94	0.70	0.15	0.56	0.69
Chhattisgarh	0.09	0.17	0.85	0.72	0.65	0.12
Jharkhand	0.00	0.00	0.77	1.00	0.77	0.00
Uttar Pradesh	0.70	0.92	0.53	0.18	0.68	0.80
Andhra Pradesh	0.32	0.32	0.77	0.77	0.65	0.32
Haryana	0.97	0.99	0.00	0.00	0.97	0.98
Orissa	0.30	0.29	0.81	0.81	0.70	0.30
Punjab	0.90	1.00	0.00	0.00	0.90	0.95
West Bengal	0.72	0.77	0.63	0.56	0.69	0.75
India	0.88	0.74	0.30	0.52	0.70	0.80

Table S6 Continued

State	G-P500	G-U500	G-NP500	G-NU500	G-AllGW500	[illegible]-F1score50[illegible]
Bihar	1.00	0.91	0.00	0.00	0.91	0.95

Chhattisgarh	1.00	0.25	0.00	0.93	0.25	0.39
Jharkhand	0.00	0.00	0.00	0.00	0.00	0.00
Uttar Pradesh	1.00	0.90	0.00	0.00	0.90	0.95
Andhra Pradesh	1.00	0.24	0.00	0.84	0.24	0.38
Haryana	1.00	1.00	0.00	0.00	1.00	1.00
Orissa	0.99	0.24	0.02	0.89	0.25	0.39
Punjab	1.00	1.00	0.00	0.00	1.00	1.00
West Bengal	1.00	0.66	0.03	0.97	0.66	0.79
India	1.00	0.70	0.01	0.93	0.70	0.82

Table S6 Continued

State	P-P500	P-U500	P-NP500	P-NU500	P-AllGW500	-F1score50
Bihar	0.64	0.95	0.64	0.15	0.64	0.77
Chhattisgarh	0.15	0.19	0.76	0.72	0.60	0.17
Jharkhand	0.00	0.00	0.77	1.00	0.77	0.00
Uttar Pradesh	0.79	0.92	0.40	0.18	0.75	0.85
Andhra Pradesh	0.47	0.37	0.71	0.79	0.65	0.42
Haryana	1.00	1.00	0.00	0.00	0.99	1.00
Orissa	0.40	0.28	0.73	0.82	0.66	0.33
Punjab	0.93	1.00	0.00	0.00	0.93	0.97
West Bengal	0.84	0.79	0.56	0.63	0.74	0.81
India	0.91	0.73	0.23	0.53	0.70	0.81

Table S6 Continued

State	G-P1000	G-U1000	G-NP1000	G-NU1000	G-AllGW1000	-F1score100
Bihar	1.00	0.92	0.00	0.00	0.92	0.96
Chhattisgarh	1.00	0.29	0.00	0.00	0.29	0.45
Jharkhand	0.00	0.00	0.00	0.00	0.00	0.00
Uttar Pradesh	1.00	0.91	0.00	0.00	0.91	0.95
Andhra Pradesh	1.00	0.24	0.00	0.92	0.24	0.38
Haryana	1.00	1.00	0.00	0.00	1.00	1.00
Orissa	1.00	0.27	0.00	0.00	0.27	0.43
Punjab	1.00	1.00	0.00	0.00	1.00	1.00
West Bengal	1.00	0.68	0.00	1.00	0.68	0.81
India	1.00	0.71	0.00	0.98	0.71	0.83

Table S6 Continued

State	P-P1000	P-U1000	P-NP1000	P-NU1000	P-AllGW1000	-F1score100
Bihar	0.77	0.93	0.43	0.15	0.74	0.84
Chhattisgarh	0.35	0.28	0.59	0.68	0.52	0.31
Jharkhand	0.00	0.00	0.33	1.00	0.33	0.00
Uttar Pradesh	0.85	0.92	0.32	0.18	0.80	0.89
Andhra Pradesh	0.59	0.32	0.54	0.79	0.55	0.41
Haryana	1.00	1.00	0.00	0.00	1.00	1.00
Orissa	0.64	0.36	0.59	0.82	0.60	0.46
Punjab	0.99	1.00	0.00	0.00	0.99	0.99
West Bengal	0.91	0.80	0.47	0.70	0.78	0.85
India	0.94	0.74	0.18	0.55	0.72	0.83

Table S6 Continued

State	G-P2000	G-U2000	G-NP2000	G-NU2000	G-AllGW2000	-F1score200
Bihar	1.00	0.93	0.00	0.00	0.93	0.97
Chhattisgarh	1.00	0.37	0.00	1.00	0.37	0.54
Jharkhand	1.00	0.03	0.00	0.00	0.03	0.06
Uttar Pradesh	1.00	0.91	0.00	0.00	0.91	0.95
Andhra Pradesh	1.00	0.26	0.00	1.00	0.26	0.41

Haryana	1.00	1.00	0.00	0.00	1.00	1.00
Orissa	1.00	0.29	0.00	0.00	0.29	0.45
Punjab	1.00	1.00	0.00	0.00	1.00	1.00
West Bengal	1.00	0.72	0.00	1.00	0.72	0.84
India	1.00	0.73	0.00	1.00	0.73	0.84

Table S6 Continued

State	P-P2000	P-U2000	P-NP2000	P-NU2000	P-AllGW2000	F1score200
Bihar	0.86	0.93	0.21	0.11	0.81	0.89
Chhattisgarh	0.67	0.39	0.37	0.64	0.48	0.50
Jharkhand	0.00	0.00	0.25	1.00	0.25	0.00
Uttar Pradesh	0.93	0.91	0.17	0.22	0.85	0.92
Andhra Pradesh	0.75	0.28	0.32	0.78	0.44	0.41
Haryana	1.00	1.00	0.00	0.00	1.00	1.00
Orissa	0.77	0.35	0.38	0.79	0.50	0.48
Punjab	1.00	1.00	0.00	0.00	1.00	1.00
West Bengal	0.96	0.80	0.35	0.77	0.80	0.87
India	0.97	0.74	0.11	0.55	0.73	0.84

Table S6 Continued

State	G-P3000	G-U3000	G-NP3000	G-NU3000	G-AllGW3000	F1score300
Bihar	1.00	0.93	0.00	0.00	0.93	0.96
Chhattisgarh	1.00	0.40	0.00	1.00	0.40	0.57
Jharkhand	1.00	0.00	0.00	0.00	0.00	0.00
Uttar Pradesh	1.00	0.93	0.00	0.00	0.93	0.96
Andhra Pradesh	1.00	0.26	0.00	0.00	0.26	0.42
Haryana	1.00	1.00	0.00	0.00	1.00	1.00
Orissa	1.00	0.32	0.00	0.00	0.32	0.49
Punjab	1.00	1.00	0.00	0.00	1.00	1.00
West Bengal	1.00	0.73	0.00	0.00	0.73	0.84
India	1.00	0.73	0.00	1.00	0.73	0.84

Table S6 Continued

State	P-P3000	P-U3000	P-NP3000	P-NU3000	P-AllGW3000	F1score300
Bihar	0.92	0.95	0.19	0.11	0.88	0.93
Chhattisgarh	0.89	0.46	0.23	0.72	0.51	0.61
Jharkhand	0.91	0.33	0.21	0.85	0.42	0.49
Uttar Pradesh	0.96	0.92	0.06	0.12	0.88	0.94
Andhra Pradesh	0.82	0.29	0.18	0.71	0.37	0.43
Haryana	1.00	1.00	0.00	0.00	1.00	1.00
Orissa	0.88	0.35	0.18	0.75	0.41	0.50
Punjab	1.00	1.00	0.00	0.00	1.00	1.00
West Bengal	0.98	0.78	0.28	0.84	0.79	0.87
India	0.98	0.74	0.06	0.53	0.73	0.84

Table S6 Continued

State	G-P5000	G-U5000	G-NP5000	G-NU5000	G-AllGW5000	F1score500
Bihar	1.00	0.97	0.00	0.00	0.97	0.98
Chhattisgarh	1.00	0.52	0.00	1.00	0.52	0.69
Jharkhand	1.00	0.20	0.00	0.00	0.20	0.33
Uttar Pradesh	1.00	0.93	0.00	0.00	0.93	0.96
Andhra Pradesh	1.00	0.28	0.00	0.00	0.28	0.44
Haryana	1.00	1.00	0.00	0.00	1.00	1.00
Orissa	1.00	0.41	0.00	0.00	0.41	0.59
Punjab	1.00	1.00	0.00	0.00	1.00	1.00
West Bengal	1.00	0.76	0.00	0.00	0.76	0.86

India	1.00	0.77	0.00	1.00	0.77	0.87

Table S6 Continued

State	P-P5000	P-U5000	P-NP5000	P-NU5000	P-AllGW5000	F1score500
Bihar	0.96	0.95	0.13	0.13	0.91	0.95
Chhattisgarh	1.00	0.48	0.06	1.00	0.49	0.64
Jharkhand	1.00	0.54	0.36	1.00	0.64	0.70
Uttar Pradesh	1.00	0.93	0.00	0.00	0.93	0.96
Andhra Pradesh	0.90	0.30	0.03	0.35	0.31	0.45
Haryana	1.00	1.00	0.00	0.00	1.00	1.00
Orissa	1.00	0.39	0.07	1.00	0.42	0.56
Punjab	1.00	1.00	0.00	0.00	1.00	1.00
West Bengal	1.00	0.81	0.16	1.00	0.81	0.89
India	0.99	0.77	0.02	0.46	0.77	0.87
